# Mononuclear Molybdenum and Tungsten Phosphine Complexes for Catalytic Ammonia Synthesis: Development of the pentaPod Concept

**DOI:** 10.1002/cphc.202500740

**Published:** 2025-12-12

**Authors:** Anna‐Marlene Vogt, Tobias Adrian Engesser, Jan Krahmer, Felix Tuczek

**Affiliations:** ^1^ Institut für Anorganische Chemie Christian‐Albrechts‐Universität zu Kiel Max‐Eyth‐Straße 2 24118 Kiel Germany

**Keywords:** ammonia, catalysis, nitrogen fixation, phosphines, tungsten

## Abstract

The pentaPod (P5) concept, combining tridentate and tripodal ligand fragments, is developed to obtain chemocatalytic Chatt‐type complexes with greater stability than classical molybdenum and tungsten systems. In these pentaPod complexes, side reactions that usually inhibit catalysis in classic Chatt complexes are effectively suppressed. Using the original pentaPod ligand P5^*Me*^, molybdenum and tungsten dinitrogen complexes [M(N_2_)(P5^*Me*^)] (M = Mo and W) are synthesized. Indeed, [Mo(N_2_)(P5^*Me*^)] generates 26 equivalents of ammonia with the PCET (*proton coupled electron transfer*) reagent SmI_2_(THF)_2_/H_2_O as electron and proton source, whereas [W(N_2_)(P5^*Me*^)] affords 3 equivalents of ammonia, but primarily catalyzes the hydrogen evolution reaction (HER). Despite their different reactivities, both complexes exhibit similar redox potentials, and DFT calculations of the mechanisms of N_2_‐to‐NH_3_ reduction and HER show no differences between [Mo(N_2_)(P5^*Me*^)] and [W(N_2_)(P5^*Me*^)]. To improve the catalytic activity of the pentaPod complexes, the modified pentaPod ligand P5^*Pln*^, containing two phospholane groups, is developed. The corresponding [M(N_2_)(P5^*Pln*^)] complexes (M = Mo and W) produce 22 (Mo) and 7 (W) equivalents of NH_3_, respectively, rendering the latter the first tungsten complex to chemocatalytically generate ammonia. Surprisingly, spectroscopic and electrochemical data indicate lower Brønsted basicities of the tungsten dinitrogen complexes compared to their molybdenum analogs.

## Graphical abstract

The pentaPod (P5) concept unites tridentate and tripodal fragments to form molybdenum and tungsten catalytic Chatt‐type complexes for N_2_‐to‐NH_3_ reduction. Two pentaPod ligands, P5^*Me*^ and P5^*Pln*^, are available with either two dimethylphosphine or phospholano (Pln) groups. The complexes enable the investigation of reactivity differences in N_2_ reduction and, in particular, allow investigation of the basicity differences between molybdenum and tungsten complexes.

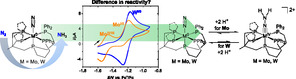

## 1 Introduction

As a fundamental constituent of amino acids and nucleobases, nitrogen is essential for the formation of proteins and DNA.^[^ ^1^, ^2^ ^]^ Consequently, converting dinitrogen (N_2_) into a bioavailable form as ammonia (NH_3_) represents a crucial reaction in nature as well as in industry and scientific research.^[^ ^3^, ^4^, ^5^, ^6^ ^]^ The most challenging aspect of this transformation is the activation of N_2_ in order to overcome the bond dissociation energy of 946 kJ mol^−1^ of the N≡N triple bond. This requires a reducing ability that surpasses the electrochemical window of water and is unattainable by any known biological reductant.^[^ ^2^, ^7^, ^8^ ^]^ In nature, the conversion of N_2_ to NH_3_ under ambient conditions takes place in diazotrophic bacteria which possess the enzyme nitrogenase.^[^ ^1^, ^8^, ^9^, ^10^ ^]^ Under ideal conditions the overall stoichiometry of the N_2_‐to‐NH_3_ reduction (N_2_RR) by the nitrogenase follows Equation (1)^[^ ^11^ ^]^

(1) $$N_{2}+8\;H^{+}+8\;e^{-}+16\;\text{MgATP}\to 2\;{\text{NH}}_{3}+H_{2}+16\;\text{MgADP}+16\;P_{i}$$

**Figure 1:**
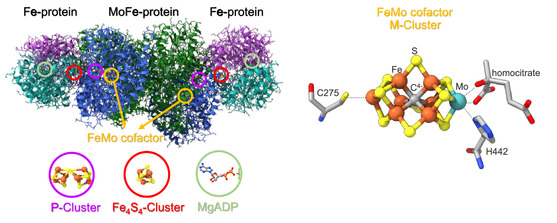
Nitrogenase complex (left) consisting of two Fe proteins and two MoFe proteins. The P and M clusters of the MoFe protein as well as the Fe_4_S_4_ cluster of the Fe protein are highlighted (PBD file: 1N2C); X‐ray structure of the FeMo‐cofactor (right), the active site of the MoFe‐protein (PBD: 1N2C; 3U7Q).^[^ ^15^ ^]^

The transformation of N_2_ to NH_3_ proceeds via successive transfer of protons (H^+^) and electrons (e^−^).^[^ ^5^, ^7^, ^9^ ^]^ The most common and well‐studied form of this enzyme is the FeMo‐nitrogenase, which is composed of the iron (Fe) and the molybdenum–iron (MoFe) protein (**Figure** **1**).^[^ ^7^, ^11^, ^12^ ^]^

The iron protein contains one Fe_4_S_4_ cluster which supplies the electrons needed for the reduction of N_2_ and an adenosine triphosphate (ATP) binding site (Figure 1).^[^ ^2^, ^13^, ^14^ ^]^ The hydrolysis of ATP increases the thermodynamic driving force for electron transfer to the MoFe protein (see above), which occurs in a complex formed by the MoFe and the Fe protein (Figure 1).^[^ ^2^ ^]^ After each electron transfer, this complex dissociates again. The MoFe‐protein, on the other hand, contains two unique iron‐sulfur clusters. In 1992, Kim and Rees published the crystal structure of the FeMo‐cofactor (FeMoco; MoFe_7_S_9_C‐homocitrate).^[^ ^11^ ^]^ The FeMoco consists of an Fe_3_MoS_3_ and an Fe_4_S_3_ part which are connected by three bridging sulfur (μ‐S) atoms. At the center of this structure, a carbidic C‐atom (C^4−^) is located (Figure 1).^[^ ^15^, ^16^, ^17^ ^]^ The bonding of N_2_ and its conversion to NH_3_ occur at the iron atoms of the FeMoco which therefore is the active site of nitrogenase. The second metallocluster within the MoFe‐protein is the P‐cluster (an Fe_8_S_7_ structure). It plays a role in electron transfer from the [Fe_4_S_4_]^+^‐cluster of the Fe‐Protein to the MoFe‐protein.^[^ ^18^, ^19^ ^]^

In 1984, Lowe and Thorneley (LT) developed a model for the kinetics of N_2_RR mediated by the enzyme nitrogenase involving eight electron‐ and eight proton‐transfer steps. Correspondingly, the LT‐model is based on eight intermediates denoted E_0_ to E_7_.^[^ ^18^, ^20^ ^]^ While six electrons are required for the nitrogen reduction reaction (N_2_RR) alone, the remaining two electrons in this process are consumed for the stoichiometric formation of H_2_ which is assumed to be associated with the binding and activation of the inert substrate N_2_.^[^ ^2^, ^13^, ^14^ ^]^

Up to the beginning of the 20th century, agriculture was more or less limited by biological nitrogen fixation, until the development of the heterogeneously catalyzed Haber–Bosch process enabled a large‐scale industrial ammonia production starting from N_2_.^[^ ^5^, ^9^ ^]^ A third way of transforming N_2_ to ammonia is synthetic nitrogen fixation which is based on transition‐metal complexes in homogeneous solution. Research on synthetic nitrogen fixation only began in the 1960s and initially focused on the design and investigation of transition‐metal complexes binding N_2_ and mediating its conversion to ammonia, analogous to the FeMo cofactor of nitrogenase.^[^ ^21^, ^22^ ^]^ This research has witnessed an impressive development in the last decades.^[^ ^5^, ^16^, ^22^, ^23^ ^]^ Nowadays, a fundamental understanding of the activation of the N_2_ molecule as well as its catalytic functionalization has been achieved, not only to NH_3_, but also to its derivatives and other nitrogen‐containing compounds.^[^ ^1^, ^5^, ^6^ ^]^ Achieving nitrogen‐containing compounds such as alkyl amines, however, remains a formidable challenge, as it is inherently difficult to activate N_2_ and a simple alkene simultaneously.^[^ ^24^ ^]^ Synthetic nitrogen fixation therefore involves not only the activation of the N_2_ molecule but also the subsequent formation of an X—N bond, with X = C in case of alkyl amines, which demonstrates great importance of this research.^[^ ^24^, ^25^, ^26^ ^]^

**Figure 2:**
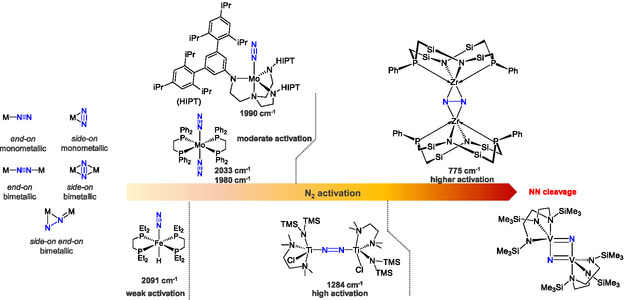
Binding modes of the N_2_ ligand in mono‐ and bimetallic complexes (left) and degree of N_2_ activation in different transition metal N_2_ complexes (right).^[^ ^27^, ^28^ ^]^

A key aspect of this progress in synthetic nitrogen fixation is to activate the highly inert N_2_ molecule for protonation. This means that negative charge has to be transferred to (at least one of) its atoms.^[^ ^27^ ^]^ However, N_2_ is difficult to polarize due to its tightly bound *σ* and *π* electrons. Moreover, coordinated N_2_ represents a poor *σ* donor and *π* acceptor.^[^ ^27^ ^]^ To obtain a systematic understanding of these factors, many dinitrogen complexes with nearly all transition metals have been synthesized in the past. The character of the metal–N bond is influenced by the metal center, its oxidation state, and the nature of the additional ligands.^[^ ^27^, ^28^ ^]^ The NN stretching frequency serves as a highly sensitive indicator of the extent of N_2_ ligand activation (**Figure** **2**).^[^ ^28^ ^]^

In the following sections, we report the development of the pentaPod concept for synthetic nitrogen fixation. To this end, we first describe the synthesis of Chatt‐type molybdenum and tungsten dinitrogen complexes supported by pentaPod ligands. Their comprehensive characterization by NMR spectroscopy and cyclic voltammetry (CV) is presented, as well as their catalytic activity in the nitrogen reduction reaction (N_2_RR). Furthermore, a possible mechanistic pathway of N_2_ reduction based on these systems is proposed and discussed. It is shown that the pentaPod framework provides a unique platform that allows the detailed and systematic investigation of N_2_ activation and catalytic conversion to NH_3_ by tuning the steric and electronic properties of the donor groups.

## 2 Chatt‐Type Molybdenum and Tungsten Complexes and the Chatt Cycle

In the 1970s, groups of Chatt and Hidai investigated the stepwise protonation and reduction of molybdenum(0) and tungsten(0) complexes of the type [M(N_2_)_2_(diphos)_2_] with M = Mo, W; diphos = depe (bis(diethylphosphino)ethane) and dppe (bis(diphenylphosphino)ethane).^[^ ^29^, ^30^, ^31^ ^]^ Molybdenum was frequently employed as a metal center in dinitrogen complexes owing to its presence in the FeMo cofactor and its assumed role, at the time, in the mechanism of nitrogenase.^[^ ^32^ ^]^ However, it is also today frequently employed in N_2_RR studies.^[^ ^2^ ^]^ Tungsten, the heavier homologue, serves as an alternative because of its comparable N_2_ coordination chemistry, in which the N_2_ ligand typically exhibits a higher activation as a result of the more electron‐releasing metal center.^[^ ^16^ ^]^ Activation of these bis(dinitrogen) complexes is typically monitored by infrared and Raman spectroscopy through their N—N stretching frequencies which show a moderately activated N_2_ ligand.^[^ ^28^, ^29^, ^33^ ^]^

Based on the mentioned Mo and W dinitrogen complexes, ammonia was generated (in generally sub‐stoichiometric amounts) by protonation with mineral acids. Chatt and Coworkers investigated the ability of complexes with monodentate phosphine ligands of the type *cis*‐[M(N_2_)_2_(PMe_2_Ph)_4_] and *trans*‐[M(N_2_)_2_(PMe_2_Ph)_4_] with M = Mo, W to generate ammonia. The highest ammonia yield (90%) was achieved using *cis*‐[W(N_2_)_2_(PMe_2_Ph)_4_] in methanol.^[^ ^33^, ^34^ ^]^ On the other hand, protonolysis of classic Chatt complexes supported by bidentate phosphines tends to stop at the stage of hydrazido(2*‐*) complexes.^[^ ^35^ ^]^ A big step forward was the first cyclic generation of ammonia using [W(N_2_)_2_(dppe)_2_], which was initially protonated by p‐toluenesulfonic acid (TsOH • H_2_O) to form the NNH_2_ complex [W(NNH_2_)(TsO)(dppe)_2_]^2+^, followed by the electrochemical reduction of this complex at a mercury pool electrode.^[^ ^36^ ^]^ Further stoichiometric addition of TsOH • H_2_O enabled two additional cycles. This procedure in total yielded 0.73 mol of ammonia, based on [W(NNH_2_)(TsO)(dppe)_2_]^2+^, with regeneration of the starting complex [W(N_2_)_2_(dppe)_2_] after three cycles.^[^ ^36^, ^37^ ^]^

In the Chatt cycle, the cyclic conversion of N_2_ to NH_3_ starts through addition of inorganic acids (HCl, HBF_4_, H_2_SO_4_) followed by an exchange of one N_2_ ligand by the conjugate base of the used acid.^[^ ^29^, ^31^, ^33^ ^]^ Along the (conceptually cyclic) pathway for N_2_RR followed by these Chatt complexes, several intermediates such as diazenido(−) M(NNH),^[^ ^38^ ^]^ hydrazido(2*‐*) M(NNH_2_),^[^ ^39^, ^40^, ^41^, ^42^, ^43^, ^44^, ^45^, ^46^ ^]^ hydrazidium M(N_2_H_3_),^[^ ^44^, ^46^, ^47^, ^48^ ^]^ and hydrazido(1*‐*) complexes M(NHNH_2_)^[^ ^49^ ^]^ were isolated or specifically synthesized and characterized by Chatt and Hidai and their coworkers. The proposed mechanism, involving (more or less, not strictly) alternating electron (ET) and proton transfer (PT) steps, indicates that the distal nitrogen is protonated initially, followed by N—N cleavage and release of NH_3_. In the second part of the cycle, the stepwise protonation of the proximal nitrogen occurs,^[^ ^50^ ^]^ leading to nitrido M(N),^[^ ^51^ ^]^ imido M(NH), and amido complexes M(NH_2_) some of which were isolated and characterized as well.

**Scheme 1:**
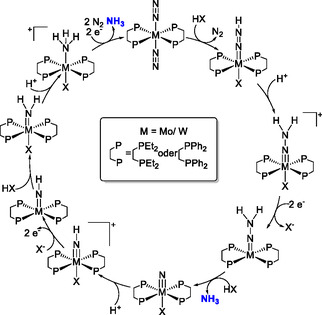
Distal pathway of the Chatt cycle; N_2_‐to‐NH_3_ conversion by [M(N_2_)_2_(diphos)_2_] M = Mo, W; diphos = depe (bis(diethylphosphino)ethane), dppe (bis(diphenylphosphino)ethane.

In the first half of the Chatt cycle, the NN bond order decreases from approximately three to one through gradual protonation. Ultimately, the N—N bond is cleaved, leading to the release of one NH_3_ molecule. At the same time, the metal–N_*α*_ bond in the complex is strengthened to a triple bond. These transformations have been confirmed by vibrational spectroscopy studies and UV/vis absorption spectroscopies as well as DFT calculations.^[^ ^9^, ^52^ ^]^ After one equivalent of NH_3_ is formed, a nitrido complex forms which can be protonated to obtain a second equivalent of NH_3_ (see above and **Scheme** **1**).^[^ ^2^, ^9^, ^16^ ^]^

In addition to experimental investigations of the Chatt cycle, computational methods were employed to evaluate the free reaction enthalpy for each protonation and reduction step throughout the cycle. In these DFT calculations, decamethylchromocene (Cp*_2_Co) was employed as the reductant, while protonation was modeled using HBF_4_/diethylether. Notably, the initial protonation of the N_2_ complex was identified as the most endothermic step, whereas NN bond cleavage emerged as the most exothermic reaction within the cycle.^[^ ^50^ ^]^

Due to the relative softness of phosphine donors, metal–ligand bonding in these systems is significantly weakened in higher‐valent intermediates, such as M^IV^ nitrido complexes.^[^ ^16^ ^]^ Another inherent issue within the Chatt cycle is the disproportionation of two [MX(N_2_)(diphos)_2_] intermediates, which results in the loss of 50% of the potential catalyst per cycle and the formation of [MX_2_(diphos)_2_] and [M(N_2_)(diphos)_2_]. To overcome this problem, it is essential to occupy the *trans* position relative to the coordinated N_2_ ligand, which plays a pivotal role in the complex's reactivity.^[^ ^1^, ^16^ ^]^

## 3 The pentaPod Concept

### 3.1 A Mo^0^ Dinitrogen Complex [MoN_2_L] (L = pentaPod, P5) with a Pentadentate Phosphine Ligand

**Scheme 2:**
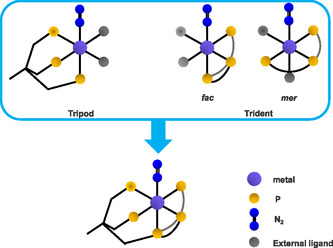
Illustration of the pentaPod concept, combining structural features of a tripodal and a tridentate ligand through a central phosphorus donor.

In our research group, two complementary strategies have been pursued to address the fundamental limitations of the classical Chatt complexes: i) employing multidentate ligands to enhance the stability of the potential catalyst for N_2_‐to‐NH_3_ conversion, and ii) saturating the position *trans* to the N_2_ ligand to suppress disproportionation reactions. Ultimately, these studies led to a pentadentate tetrapodal phosphine ligand (pentaPod, P5) that coordinates the metal center in pseudo‐4‐fold symmetry and enables the binding and reduction of N_2_ at a single coordination site in *trans* position to the focal P donor. This ligand type features a topology that merges the features of a tripodal and a tridentate ligand, connected at the central phosphorus donor of the tridentate part (**Scheme** **2**).^[^ ^53^ ^]^

**Scheme 3:**
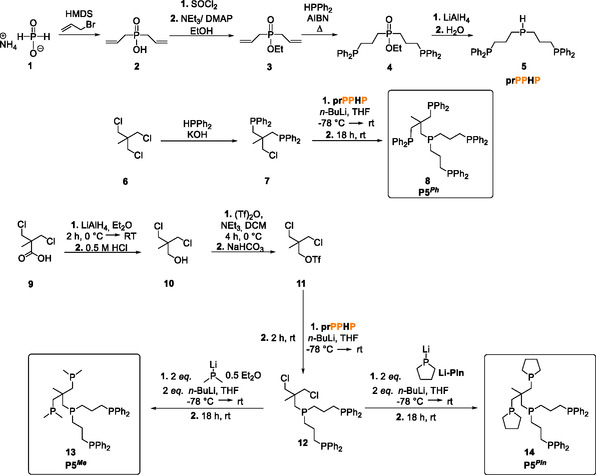
Synthesis of the symmetric pentaPod ligand P5^*Ph*^ (**8**) and asymmetric P5^*Me*^ (**13**) and P5^*Pln*^ (**14**).

At the outset of developing the pentaPod concept, we focused on two distinct pentaPod ligands: one symmetrically substituted P5^*Ph *^(**8**), bearing four diphenylphosphine end groups, and one asymmetrically substituted P5^*Me*^ (**13**), featuring dimethylphosphine groups in the tripodal segment and diphenylphosphine groups in the tridentate part (see **Scheme** **3**). Initially, the focus was primarily on molybdenum complexes. Therefore, coordination of the ligand to the precursor [MoCl_3_(thf)_3_] (**15**), followed by reduction with sodium amalgam, was expected to yield M^0^ dinitrogen complexes.^[^ ^53^ ^]^ Starting from ammonium phosphinate (**1**), the tridentate part of the pentaPod ligands **5** was obtained through a four‐step synthesis (Scheme 3).^[^ ^53^, ^54^, ^55^ ^]^

Further steps for the synthesis of the symmetrically substituted P5^*Ph*^ (**8**) ligand proceeded according to Muth et al. by deprotonating 1,3‐dichloro‐2‐(chloromethyl)propane (**6**) with KOH, followed by reaction with two equivalents of KPPh_2_ to give the ligand fragment **7** with the desired tripodal structure.^[^ ^56^ ^]^ Subsequent treatment with in situ lithiated phosphine species **5**, obtained after adding *n*‐BuLi to **5** in THF, afforded P5^*Ph*^. Synthesizing the asymmetric ligand P5^*Me*^ (**13**) proved more demanding. For this purpose, 3,3′‐dichloropivalic acid (**9**) was converted to the selectively activated building block chlorido alkyl‐triflate (**11**) in two synthetic steps. Again, the in situ lithiated species **5** reacted with **11** to give compound **12**. Finally, treatment of **12** with two equivalents of LiPMe_2_ • 0.5 Et_2_O yielded the asymmetric ligand P5^*Me*^ (**13**) (see Scheme 3).^[^ ^53^ ^]^ The synthesis and characterization of pentaPod ligand P5^*Pln*^ (**14**) followed the same approach as for the first pentaPod ligand **13**. In the final step, P5^*Pln*^ (**14**) was synthesized using Li‐Pln instead of LiPMe_2_ • 0.5 Et_2_O in the final step.^[^ ^57^ ^]^

Our study initially focused on complexes supported by the symmetric pentaPod ligand P5^*Ph*^ (**8**) and the asymmetric ligand P5^*Me*^ (**13**). Subsequently, we examined the second asymmetric pentaPod ligand P5^*Pln*^ (**14**) and the complexes derived from it (see section 3.4).

The coordination behavior of P5^*Ph*^ (**8**) showed that symmetric pentaPod ligands are not suitable for forming well‐defined Mo^0^ dinitrogen complexes. Reaction of ligand **8** with [MoCl_3_(thf)_3_] gave a product with the formula [MoCl_3_(P5^*Ph*^)]. However, amalgam reduction under N_2_ afforded a product with several IR bands in the spectral region of N—N absorption, indicating that no defined mononuclear Mo^0^ dinitrogen species had formed.^[^ ^53^ ^]^

In contrast, after the reaction of P5^*Me*^ (**13**) with the precursor [MoCl_3_(thf)_3_], the complex *fac(trpd)*‐[MoCl_3_(P5^*Me*^)] was first obtained and characterized by EPR. The tripodal (*trpd*) part of the pentaPod ligand **13** selectively coordinates first to the molybdenum center due to the lower steric demand and stronger σ‐donor character of the alkylphosphine donors in the tripodal part of P5^*Me*^ (**13**). This shows that the nucleophilicity of the different phosphine donors is crucial for the coordination of a pentaPod ligand. Sodium amalgam reduction of the Mo^III^ precursor **16** coordinated to the asymmetrically substituted ligand **13** afforded the desired mononuclear dinitrogen complex [Mo(N_2_)(P5^*Me*^)] (**17**).^[^ ^53^ ^]^

**Scheme 4:**
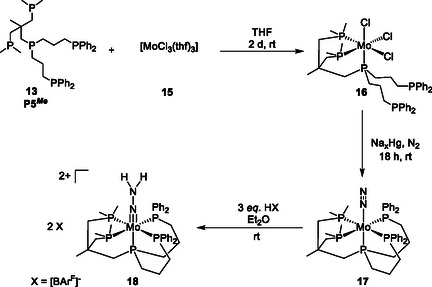
Synthesis of *fac(trpd)*‐[MoCl_3_(P5^*Me*^)] (**16**) and subsequent sodium amalgam reduction results in formation of the desired [Mo(N_2_)(P5^*Me*^)] (**17**). Protonation of **17** with three equivalents of the acid [Et_2_O)_2_H][BAr^F^] (BAr^F^ = tetrakis(3,5‐bis(trifluoromethyl)phenyl)borate) gives the corresponding NNH_2_ complex **18**.

Following the example of Chatt and coworkers, we also wanted to protonate complex [Mo(N_2_)(P5^*Me*^)] (**17**) and therefore explored the reactivity of this complex toward acids with weakly coordinating anions such as [(Et_2_O)_2_H][BAr^F^] (BAr^F^ = tetrakis(3,5‐bis(trifluoromethyl)phenyl)borate). The structure of the coordinated pentaPod should prevent an unwanted coordination of the counter anion in the formed cationic complex as observed for the classic Chatt‐type NNH_2_ complexes such as [MoF(NNH_2_)(diphos)_2_].^[^ ^41^ ^]^ Using less than three equivalents of acid did not yield the desired product [Mo(NNH_2_)(P5^*Me*^)][BAr^F^]_2_ (**18**) (**Scheme** **4**).^[^ ^58^ ^]^

**Figure 3:**
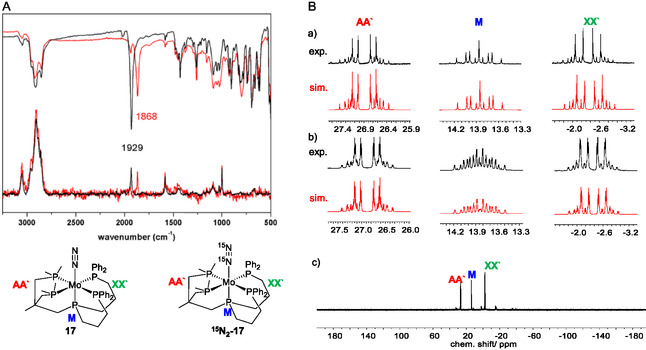
A) Adapted with permission from ref. [53]. Copyright 2016, American chemical Society: IR and Raman spectrum of [Mo(N_2_)(P5^*Me*^)] (**17**) (black) and [Mo(^15^N_2_)(P5^*Me*^)] (^**15**^ **N**_**2**_ **‐17**) (red); B) ^31^P{^1^H} NMR spectra of **17** (a,c; black) and ^**15**^ **N**_**2**_ **‐17** (b) measured in benzene –*d*_6_ at 300 K, in comparison with the simulations (red).^[^ ^53^ ^]^

As expected, the IR and Raman spectrum of **17** shows one defined absorption band that can be assigned to the NN stretching vibration, confirming the formation of a mononuclear Mo^0^ dinitrogen complex. Remarkably, the observed band at ν(NN) = 1929 cm^−1^ (**Figure** **3**A) represents the lowest reported N—N stretch for mononuclear Mo^0^ dinitrogen complexes with a pentaphosphine environment.^[^ ^53^, ^59^ ^]^ N_2_ activation depends on the electron density at the Mo^0^ center, which is influenced by the type of phosphine donors. Comparison with other mono dinitrogen complexes, which also have a pentaphosphine environment containing PMe_2_ and PPh_2_ donors in the equatorial plane but different phosphines in axial position (P_ax_), showed that N_2_ activation strongly depends on P_ax_,^[^ ^60^ ^]^ that is, with increasing electron‐donating ability of P_ax_ in the order PPh_3_ < PRH < PR_3_ (R = alkyl), the activation of the N_2_ ligand also increases.^[^ ^60^, ^61^ ^]^ To gain more insight into the electronic properties of **17**, N_2_ activation and the coupling between the phosphorus atoms of the pentaPod ligand **13** and the N atoms of coordinated N_2_, an isotopically labeled complex was prepared. Correspondingly, under ^15^N_2_‐atomsphere, the sodium amalgam reduction of the Mo^III^ precursor **16** resulted in the formation of [Mo(^15^N_2_)(P5^*Me*^)] (^**15**^ **N**_**2**_ **‐17**). For this complex a NN‐stretch at ν(NN) = 1868 cm^−1^ was observed (Figure 3A).^[^ ^53^ ^]^

The ^31^P{^1^H} NMR of [Mo(N_2_)(P5^*Me*^)] (**17**) shows the characteristic AA′XX′M splitting pattern of a pentaphosphine ligation with AA’ assigned to the PPh_2_ groups, XX′ to the PMe_2_ groups, and the M signal corresponding to P_ax_.^[^ ^53^, ^60^ ^]^ Simulation of the ^31^P{^1^H} spectrum enabled determination of the metal‐mediated coupling constants, yielding a *trans* coupling of the equatorial phosphine donors (P_AA′_ and P_XX′_) of *J*_trans_ = 83 Hz and *cis* couplings ranging from −19 < *J*_cis_ < 29 Hz. In case of [Mo(^15^N_2_)(P5^*Me*^)] (^**15**^ **N**_**2**_ **‐17**), the expected additional signal splitting is observed as a result of coupling between the ^15^N and ^31^P nuclei (Figure 3B). Notably, the M signal of P_ax_ shows significantly stronger couplings (^*2*^ *J*(^*31*^ *P*, ^*15*^ *N*_*α*_) = 13.5 Hz; ^3^ *J*(^31^ *P*, ^15^ *N*_*β*_) = 1.4 Hz) than the *cis*‐positioned equatorial phosphines PPh_2_ (AA′) and PMe_2_ (XX′), respectively (^2^ *J*(^31^ *P*_A_, ^15^ *N*_*α*_) = 3.1 Hz; ^3^ *J*(^31^ *P*_A_, ^15^ *N*_*β*_) < 1.0 Hz; ^2^ *J*(^31^ *P*_X_, ^15^ *N*_*α*_) = 3.0 Hz; ^2^ *J*(^31^ *P*_X_, ^15^ *N*_*β*_) = 1.0 Hz).^[^ ^60^ ^]^ This demonstrates the significant influence of the phosphorus atom P_ax_ on the bonding of N_2_ in the *trans* position.^[^ ^60^ ^]^

The coordination of the pentaPod ligand P5^*Me*^ (**13**) to the Mo^0^ center was evidenced by a single crystal structure of **17**, in agreement with the solution structure derived from ^13^P{^1^H} NMR. The Mo–N_*α*_ bond length measures 2.033(5) Å, while the N_*α*_–N_*β*_ bond corresponds to 1.099(5) Å, which is slightly elongated compared to elemental N_2_ (1.0975 Å).^[^ ^53^ ^]^ The Mo–P_eq_ bond lengths in the equatorial plane span from 2.4433(14) to 2.4536(13) Å, whereas the Mo–P_ax_ bond *trans* to the N_2_ ligand is distinctly shorter (2.3868(12) Å), also emphasizing the strong bonding of the axial P donor which is strapped to the Mo^0^ center via three alkyl bridges.^[^ ^53^ ^]^ However, this strong bonding interaction does not lead to an elongation of the Mo—N bond in *trans*‐position, as the Mo—N distance in **17** remains at the lower end of the range (2.025(6) – 2.099(3) Å) observed for molydenum(0) dinitrogen complexes with pentaphosphine coordination. Of particular interest in this regard is the complex [Mo(N_2_)(dpepp)(dppm)] (dpepp = bis‐((diphenyl‐phosphino)ethyl)phenylphosphine; dppm = bis(diphenylphosphino)methane), which features a notably shorter Mo—N bond (2.025(6) Å), but a longer N—N bond (1.119(8) Å) compared to [Mo(N_2_)(P5^*Me*^)] (**17**). Nevertheless, the N_2_ ligand is more activated in complex **17** (1929 cm^−1^) than in [Mo(N_2_)(dpepp)(dppm)] (1972 cm^−1^), indicating that the metal–N and N—N bond lengths in these systems are not correlated in a straightforward manner with N_2_ activation.^[^ ^62^, ^63^, ^64^ ^]^

Following the characterization of [Mo(N_2_)(P5^*Me*^)] (**17**) and aiming at the final goal, the (catalytic) formation of ammonia, we investigated the protonation of **17** and explored the reactivity and stability of the corresponding NNH_2_ pentaPod complex [Mo(NNH_2_)(P5^*Me*^)][BAr^F^]_2_ (**18**) (Scheme 4).^[^ ^58^ ^]^ DFT calculations were performed to assist in the interpretation of the experimental data and clarify the bonding situation of the NNH_2_ ligand in complex **18**,^[^ ^23^ ^]^ whereas the classic Chatt complex [MoF(NNH_2_)(diphos)_2_] is best described by an isodiazene complex (Mo=N=NH_2_) DFT predicted for [Mo(NNH_2_)(P5^*Me*^)][BAr^F^]_2_ (**18**) a hydrazido(2‐) (Mo≡N‐NH_2_) configuration.^[^ ^23^, ^65^, ^66^ ^]^ The metal≡N_*α*_ triple bond is expected to exert a pronounced *trans* effect. This is also borne out by DFT calculations which reveal a significant elongation of the Mo–P_ax_ bond in complex **18** relative to molybdenum dinitrogen complex **17**. In agreement with this, the protonation‐induced high‐field shift in the ^31^P{^1^H} NMR spectrum [Mo(NNH_2_)(P5^*Me*^)][BAr^F^]_2_ (**18**) is significantly larger for the M signal than for the A and X signals.^[^ ^60^ ^]^

**Figure 4:**
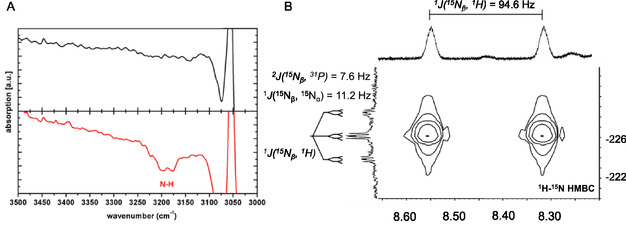
A) Adapted with permission from ref. [53]. Copyright 2016, American chemical Society: NH region of solution‐phase IR spectrum of **17** in dichloromethane (top, black) and [Mo(NNH_2_)(P5^*Me*^)][BAr^F^]_2_ (**18**) in situ generated with [(Et_2_O)_2_H][BAr^F^] (bottom, red) at 300 K; B) Enlarged region in the ^1^H‐^15^N HMBC spectrum of the N_*β*_H_2_ part of ^**15**^ **N‐17** in diethylether‐*d*_10_.^[^ ^53^, ^60^ ^]^

Protonation of **17** has been monitored with IR spectrosopy in solution. After addition of [(Et_2_O)_2_H][BAr^F^], the N—N stretch disappears and NH‐stretching vibrations appear in the expected spectral region, that is, at ≈200 cm^−1^ (**Figure** **4**, left).^[^ ^53^, ^60^ ^]^ Similar to the dinitrogen complex 17, the ^**31**^ **P{** ^**1**^ **H} NMR** spectrum of [Mo(NNH_2_)(P5^*Me*^)][BAr^F^]_2_ (**18**) displays an AA′XX′M pattern, with chemical shifts and coupling constants altered relative to those of the starting material [Mo(N_2_)(P5^*Me*^)] (**17**). This indicates the pentaphosphine environment of the pentaPod ligand P5^*Me*^ (**13**) is retained upon protonation.^[^ ^60^ ^]^ Further evidence is provided by the ^1^H‐^15^N HMBC spectrum of this complex which shows the doublet for protons and triplet for the nitrogen of the NH_2_ group in the hydrazido(2‐) ligand with a coupling constant of ^1^ *J*(^15^ *N*_*β*_,^1^ *H*) = 94.6 Hz. Additionally, couplings from N_*β*_ to N_*α*_ (11.2 Hz) and the trans standing P_ax_ (7.6 Hz) can be observed (Figure 4B).^[^ ^60^ ^]^

Inspired by the work of Nishibayashi et al., who employed the PCET (*proton coupled electron transfer*)^[^ ^67^ ^]^ reagent SmI_2_(THF)_2_/H_2_O (or SmI_2_(THF)_2_/alcohol) for N_2_ reduction, similar conditions were applied to complex **17** (see Equation 2).^[^ ^68^ ^]^ Under a dinitrogen atmosphere (1 atm) and in the presence of 180 equivalents of SmI_2_ and water in THF, complex **17** generated 25.73 ± 0.37 equivalents of ammonia.^[^ ^60^ ^]^ Thus, the complex [Mo(N_2_)P5^*Me*^] (**17**) was shown to be the first Chatt‐type system with a single coordination site capable of catalytically converting N_2_ into ammonia. Under the same conditions, the in situ generated [Mo(NNH_2_)P5^*Me*^][BAr^F^]_2_ (**18**) was also applied as catalyst and added to the solution of SmI_2_(THF)_2_/H_2_O, generating 26.14 ± 0.32 equivalents of ammonia, which is within the error limit of the Mo^0^ dinitrogen complex **17** (see Equation 2).^[^ ^60^ ^]^

(2) $$N_{2}+6\;H_{2}O+6\;{\text{Sml}}_{2}{\left(\text{THF}\right)}_{2}\;\overset{\begin{array}{c} 2\;\text{μmol}\\ \text{catalyst} \end{array}}{\underset{\text{THF,rt,10h}}{\to }}\;{\text{NH}}_{3}$$

**Figure 5:**
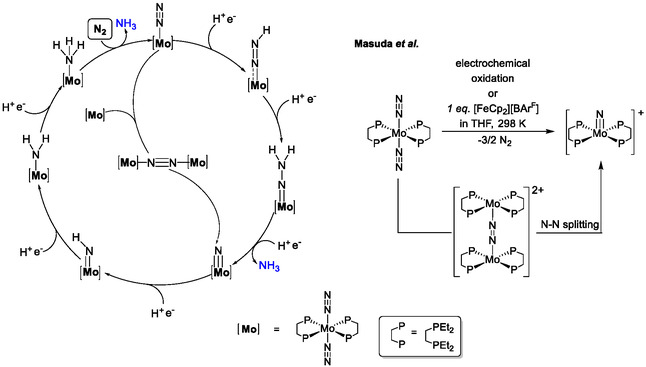
Proposed distal catalytic (Chatt) cycle for nitrogen fixation (left);^[^ ^5^, ^6^ ^]^ generation of a dinuclear Mo^I^ complex from [Mo(N_2_)_2_(depe)_2_] through one‐electron oxidation, resulting in a Mo^IV^ nitrido species (right).^[^ ^69^, ^70^ ^]^

The fact that the NNH_2_ complex **18** generated similar amounts of ammonia as its dinitrogen precursor **17** suggests that the mechanistic pathway follows the classic Chatt cycle. Due to the involvement of a PCET reagent (as opposed to the separate addition of acid and reduction), however, one electron and one proton are transferred simultaneously and the cycle has to be recast into six PCET steps (**Figure** **5** (left), outer cycle).

Whereas most of the known Chatt‐type complexes can be assumed to follow the classic Chatt cycle with a distal protonation of the N_2_ ligand, Masuda and colleagues recently provided evidence for an alternative scenario for N_2_‐to‐NH_3_ reduction by a classic Chatt‐type system, [Mo(N_2_)_2_(depe)_2_] (depe = bis(diethylphosphino)ethylene). Thereby the dinuclear Mo^I^ complex [Mo(depe)_2_]_2_(μ‐N_2_)^2+^ is formed which undergoes N—N bond cleavage, yielding two [Mo(N)(depe)_2_]^+^ cations (Figure 5 right) that can be protonated and reduced to give ammonia.^[^ ^69^, ^70^ ^]^ This way, the first half of the classic Chatt cycle is bypassed (Figure 5 (left), center). Recent studies of the Chatt complexes [W(N_2_)_2_(depe)_2_] and [W(N_2_)_2_(dppe)_2_] by Chirik et al. demonstrated that the pathway for dinitrogen splitting is highly influenced by the steric demand of the phosphine ligands in the investigated Chatt system [W(N_2_)_2_(dppe)].^[^ ^71^ ^]^ In principle, the topology of the pentaPod ligand does not allow dimerization of the catalytically active Chatt‐type catalyst **17**, which supports the assumption of a mononuclear pathway along the Chatt cycle for N_2_‐to‐NH_3_ conversion catalyzed by this system (see above).

Besides **17**, a number of classic Chatt complexes have been examined with regard to catalytic NH_3_ formation in the presence of SmI_2_/water.^[^ ^72^ ^]^ Among these, only [Mo(N_2_)_2_(PMePh_2_)_4_] exhibited a catalytic activity comparable to **17**. However, in view of the fact that this complex undergoes phosphine ligand exchange, retention of the molybdenum tetraphosphine ligation during the entire cycle is less probable. The same applies to *cis*, *mer*‐[Mo(NNH_2_)(OTf)_2_(PMePh_2_)_3_] which gave catalytic amounts of NH_3_ as well. On the other hand, when substitutionally more inert [Mo(N_2_)_2_(dppe)_2_] was employed, a drastic decrease of catalytic activity was observed. This is presumably due to ligand exchange processes at the *trans* position of the protonated N_2_ ligand which are precluded in the single‐site catalyst.

To enable an exergonic transfer of one electron and one proton on the N_2_ complex, its N—H bond dissociation free energy (N—H BDFE) must be higher than that of the PCET reagent used. An initial assessment of our system's N_2_ reduction potential was obtained by calculating the N—H BDFE of the corresponding diazenido(‐) complex via DFT. Therefore, the reaction between [Mo(N_2_)(P5^*Me*^)] (**17**) and TEMPO‐H, a hydrogen atom transfer (HAT) reagent with a well‐defined O—H bond BDFE of 65.2 kcal mol^−1^ in benzene, was considered on a theoretical level. The transfer of one electron and one proton to the Mo^0^‐dinitrogen complex **17** results in a neutral [Mo(NNH)(P5^*Me*^)] intermediate. Based on the energy required for the process TEMPO‐H → TEMPO• + H•, a N–H BDFE of Δ_r,theo_G^298^ = 19.2 kcal mol^−1^ was determined for the neutral Mo^I^‐diazenido(‐) species. This value is slightly lower than the O—H BDFE of the PCET reagent SmI_2_(THF)_2_/H_2_O (26 kcal mol^−1^),^[^ ^67^ ^]^ indicating that proton‐coupled electron transfer to the Mo(0)‐dinitrogen complex **17** is mildly endergonic (ΔG^298^ = +6.8 kcal mol^−1^), yet still thermodynamically accessible. However, referring to the classical Chatt cycle (Scheme 1) the Mo^II^‐diazenido(‐) corresponds to an intermediate within the cycle. This species would be preserved by a PCET step from a cationic Mo^I^‐dinitrogen complex. Using a similar approach as described above, the N—H BDFE for the Mo^II^‐diazenido(‐) intermediate was calculated to be Δ_r,theo_G^298^ = 52.5 kcal mol^−1^. This value significantly exceeds the BDFE of the PCET reagent SmI_2_(THF)_2_/H_2_O, indicating that proton‐coupled electron transfer to the monocationic [Mo(N_2_)(P5^*Me*^)]^+^ complex is highly exergonic (ΔG^298^ = –26.5 kcal mol^−1^).^[^ ^60^ ^]^

### 3.2 Mo versus W: Reactivity of [M(N_2_)(P5^*Me*^)] Complexes

The differing abilities of molybdenum and tungsten complexes to activate N_2_ and catalyze its conversion to NH_3_ have long attracted interest in the field of nitrogen fixation.^[^ ^5^, ^6^, ^23^, ^73^ ^]^ In several cases, attempts to prepare tungsten analogues of active molybdenum catalysts were either not successful, or if synthesis was possible they exhibited no catalytic ability for ammonia production.^[^ ^73^, ^74^ ^]^ In 2022, the Chatt‐type complex [W(N_2_)_2_(dppe)_2_] in combination with a cobalt‐based redox mediator electrocatalytically generated 11.3 ± 0.5 equivalents of ammonia.^[^ ^75^ ^]^ This is remarkable, as it shows that classical Chatt‐type tungsten complexes can in principle catalytically produce ammonia, suggesting that tungsten catalysts may also enable chemocatalytic N_2_RR.^[^ ^75^, ^76^ ^]^ However, a comparable chemocatalytically active system for N_2_RR based on tungsten has not been known at that time. Consequently, and following the example set by Chatt and Pickett, our efforts focused on synthesizing a tungsten(0) dinitrogen complex bearing the pentaPod ligand P5^*Me*^. Up to that point, [W(N_2_)(PMe_3_)_5_] had been the only reported tungsten dinitrogen complex with a pentaphosphine coordination sphere.^[^ ^77^ ^]^

**Scheme 5:**
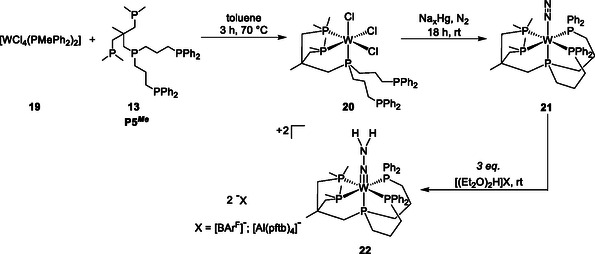
Synthesis of *fac(trpd)*‐[WCl_3_(P5^*Me*^)] (**19**) and subsequent sodium amalgam reduction to [W(N_2_)(P5^*Me*^)] (**21**). Synthesis of the corresponding hydrazido(2‐) complex [W(NNH_2_)(P5^*Me*^)][BAr^F^]_2_ (**22**) by protonation with [(Et_2_O)_2_H][BAr^F^] of **21**.^[^ ^78^ ^]^

The tungsten(III) complex **20** bearing the precoordinated pentaPod ligand was synthesized by stirring P5^*Me*^ (**13**) and [WCl_4_(PMePh_2_)_2_] (**19**) in toluene at 70 °C for 3 h. In this reaction, the released methyldiphenylphosphine serves as a reducing agent, converting tungsten(IV) to tungsten(III), while itself being oxidized to methyldiphenylphosphine dichloride. Analogous to the molybdenum(0) dinitrogen complex **17**, [W(N_2_)(P5^*Me*^)] (**21**) was prepared via sodium amalgam reduction of [WCl_3_(P5^*Me*^)] (**19**). The corresponding tungsten hydrazido(*2‐*) complex **22** was synthesized by adding three equivalents of Brookhart's acid [(Et_2_O)_2_H]X with X = [BAr^F^]^−^ or [Al(pftb)_4_]^−^ ([Al(pftb)_4_]^−^ = tetrakis(perfluoro‐*tert*‐butoxy)aluminate) (**Scheme** **5**). Similar to [Mo(NNH_2_)(P5^*Me*^)]^2+^ (**18**), smaller amounts of acid did not lead to the desired product **22**.^[^ ^78^ ^]^

**Figure 6:**
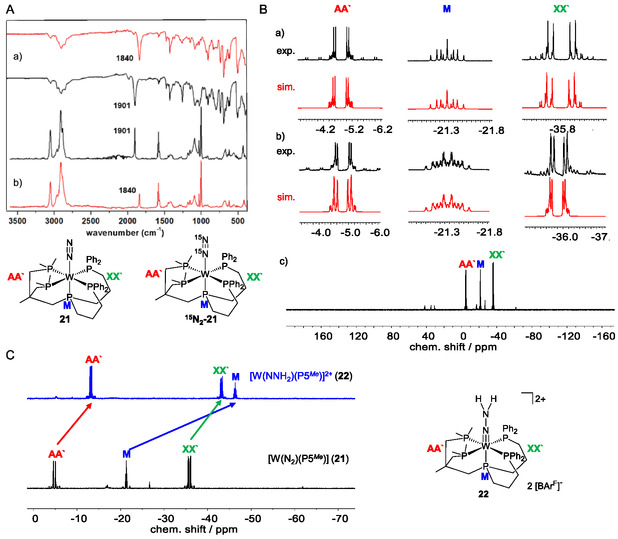
A) a) IR and b) Raman spectrum of the complex **21** (black) and ^**15**^ **N**_**2**_ **‐21** (red); B) ^31^P{^1^H} NMR spectra of **21** (a,c; black) and ^**15**^ **N**_**2**_ **‐21** b) measured in benzene‐*d*_6_ at 300 K, in comparison with the simulations (red); C) comparison of the ^31^P{^1^H} NMR spectra of **22‐BAr** ^**F**^ (blue) and **21** (black).^[^ ^78^ ^]^

Analysis of the IR spectrum of [W(N_2_)(P5^*Me*^)] (**21**) revealed the N—N stretching vibration at 1901 cm^−1^ which shifts to 1840 cm^−1^ in the ^15^N‐labeled complex [W(N_2_)(P5^*Me*^)] (^**15**^ **N**_**2**_ **‐21**). Importantly, this stretching frequency is lower than the *ν*(NN) observed for the Mo analogue **17** (1929 cm^−1^) (**Figure** **6**A). The ^31^P{^1^H} NMR spectrum of complex **21** and the corresponding hydrazido(2‐) complex **22** displayed the expected AA`XX`M pattern (Figure 6B).^[^ ^78^ ^]^^183^W satellites were observed with ^1^ *J*_W–P_ coupling constants ranging from 254 to 315 Hz, consistent with values reported in the literature for only other tungsten dinitrogen complex with a pentaphosphine environment [W(N_2_)(PMe_3_)_5_].^[^ ^77^, ^78^ ^]^

The ^31^P{^1^H} NMR spectrum of complex [W(N_2_)(P5^*Me*^)] (**21**) shows a high‐field shift between −30 and −34 ppm of all signals of the AA′XX′M coupling pattern compared to the molybdenum analogue [Mo(N_2_)(P5^*Me*^)] (**17**). Comparison of the coupling constants of these two dinitrogen complexes revealed that *cis* coupling constants are ≈10 Hz smaller for the tungsten complex **21**, while *trans* couplings are essentially unchanged, in agreement with the literature.^[^ ^78^, ^79^ ^]^ The ^15^N–^15^N and ^15^N–^31^P couplings differ by <1 Hz between complexes **21** and **17**.^[^ ^78^ ^]^ Single crystal X‐ray analysis further shows that the metal–phosphorus and N—N bond lengths of the N_2_ ligand of both complexes are almost identical,^[^ ^78^ ^]^ which means that structurally, no significant differences are observed between the [Mo(N_2_)(P5^*Me*^)] (**17**) and its heavier homologue [W(N_2_)(P5^*Me*^)] (**21**).

In the next step, the catalytic properties of the tungsten(0) dinitrogen complex **21** were examined under conditions identical to those used for [Mo(N_2_)(P5^*Me*^)] (**17**).^[^ ^60^, ^78^ ^]^ Under dinitrogen atmosphere (1 atm) and in the presence of 180 equivalents of PCET reagent SmI_2_ and water in THF, complex **21** generated 2.75 ± 0.23 equivalents of NH_3_, which corresponds to a slightly overstoichiometric conversion of N_2_ to ammonia.^[^ ^78^ ^]^ Thus, [W(N_2_)(P5^*Me*^)] (**21**) represents the first chemocatalytically active tungsten complex to produce more than two equivalents of NH_3_ per metal center upon proton and reductant addition.^[^ ^73^, ^74^ ^]^ However, this treatment also resulted in the formation of 80 equivalents of H_2_.^[^ ^80^ ^]^ In tungsten‐based systems, N_2_RR is known to be outcompeted by the hydrogen evolution reaction (HER), which also seems to be the case here.^[^ ^5^, ^6^, ^75^, ^80^ ^]^

The factors responsible for the distinct reactivity of molybdenum versus analogous tungsten complexes in N_2_RR are not understood. To gain further insight into this problem, we investigated dinitrogen complexes **21** and **17** by cyclic voltammetry and in situ IR‐spectroelectrochemistry. The electrochemical properties of the M^0^ dinitrogen pentaPod complexes (M = Mo (**17**) and W (**21**)) as well as the stabilities of their one‐electron oxidized M^I^(N_2_)^+^ counterparts are of special interest because of our DFT calculations, which we carried out for [Mo(N_2_)(P5^*Me*^)] (**17**) and the corresponding hydrazido(2‐) complex **18**.^[^ ^23^, ^60^ ^]^ These DFT calculations of the N—H BDFE for the [Mo^II^(NNH)(P5^*Me*^)]^+^ diazenido(‐) complex within a PCET framework suggest that the N_2_‐to‐NH_3_ reaction pathway could also involve a cationic, mononuclear Mol(N_2_)^+^ intermediate,^[^ ^78^ ^]^ although the molybdenum(0) dinitrogen complex **17** and the corresponding hydrazido(2‐) derivative **18** were found to be catalytically active.^[^ ^60^ ^]^ This has led us to focus particularly on the W^I^/W^0^ and Mo^I^/Mo^0^ redox couples.

**Figure 7:**
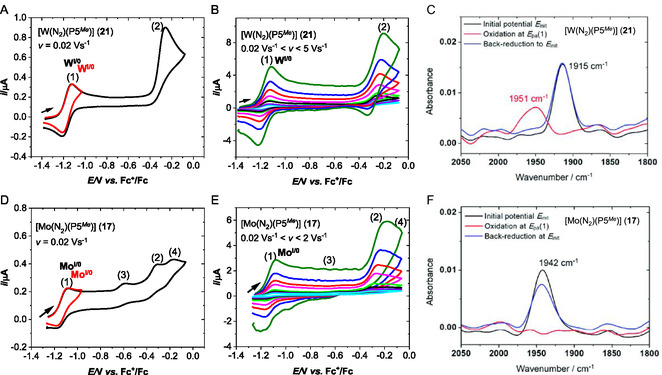
A) CVs (*E*/V Fc^+^/Fc) at a Pt working electrode (diam. 1 mm) of [W(N_2_)(P5^*Me*^)] (**21**) (0.4 mM) in THF/NaBPh_4_ (20 mM) at scan rates *v* = 0.02 V s^−1^; B) CV of **21** at different scan rates *v* = 0.02 (cyan), 0.05 (violet), 0.1 (black), 0.2 (green), 0.5 (pink), 1 (red), 2 (blue), and 5 V s^−1^ (olive); the numbers (1) and (2) on the graphics are related to the redox systems; C) infrared spectra of [W(N_2_)(P5^*Me*^)] (**21**) (15 mM) in THF/NaBPh_4_ (20 mM) measured by in situ spectroelectrochemical measurements before (black) and after oxidation at *E*_pa_(1) (red), then returning back to the initial potential (blue); D) CVs (*E*/V versus Fc^+^/Fc) at a Pt working electrode (diam. 1 mm) of [Mo(N_2_)(P5^*Me*^)] (**17**) (0.4 mM) in THF/NaBPh_4_ (20 mM) at scan rates *v* = 0.02 V s^−1^; E) CV of **17** at different scan rates *v* = 0.02 (cyan), 0.05 (violet), 0.1 (black), 0.2 (green), 0.5 (pink), 1 (red), and 2 V s^−1^ (olive); the numbers (1), (2), (3), and (4) on the graphics are related to the redox events described in Scheme 6; F) infrared spectra of [Mo(N_2_)(P5^*Me*^)] (**17**) (15 mM) in THF/NaBPh_4_ (20 mM) measured by in situ spectroelectrochemical measurements before (black) and after oxidation at *E*_pa_(1) (red), then returning back to the initial potential (blue).^[^ ^78^ ^]^

All electrochemical experiments were conducted under argon atmosphere in a solution of 20 mM NaBPh_4_ (BPh_4_ = tetraphenylborate) in dry THF as electrolyte. After confirming the stability of the dinitrogen complexes [Mo(N_2_)(P5^*Me*^)] (**17**) and [W(N_2_)(P5^*Me*^)] (**21**) under these electrochemical conditions by IR spectroscopy, cyclic voltammetry of the tungsten(0) dinitrogen complex **21** was performed at a platinum electrode across a range of scan rates (0.02 to 5 V s^−1^). Upon reduction, no cathodic peak was observed down to the potential limit of electrolyte decomposition (−2.9 V versus Fc^+^/Fc). At low to moderate scan rates (0.02 V s^−1^ < *v* < 0.2 Vs^−1^), two main redox processes were identified on the anodic side. The first appears reversible with *E*_1/2_(1) = −1.16 V, while the second is irreversible, showing an anodic peak at *E*_pa_(2) = −0.25 V versus Fc^+^/Fc (**Figure** **7**A). As the scan rate was increased up to 5 V s^−1^, the second process began to exhibit partial reversibility, whereas the first remained fully reversible (Figure 7B). This behavior suggests that the second oxidation is followed by a moderately fast (millisecond‐scale) chemical step.^[^ ^78^ ^]^

**Scheme 6:**
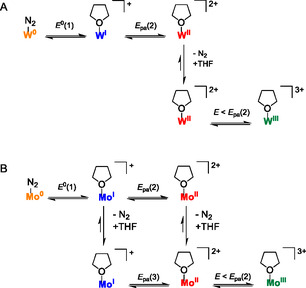
Suggested mechanisms for the redox processes observed during cyclic voltammetry of the A) tungsten and B) molybdenum pentaPod N_2_ complexes.^[^ ^78^ ^]^

To identify the species formed during the first and second oxidation events, IR spectroelectrochemical measurements were performed on [W(N_2_)(P5^*Me*^)] (**21**). Oxidation at *E*_pa_(1) led to the disappearance of the N_2_ stretching band at 1915 cm^−1^ assigned to the N_2_ ligand and the appearance of a new absorption band at 1951 cm^−1^, which can be assigned to the N—N stretch of species [W^I^(N_2_)(P5^*Me*^)]^+^ (Figure 7C). Reversing the potential fully restored the original spectrum of initial [W(N_2_)(P5^*Me*^)] (**21**), confirming the reversible nature of this redox event for the W^I^/W^0^ redox couple at *E*_1/2_(1) = −1.16 V versus Fc^+^/Fc (Figure 7C). In the second oxidation process at *E*_pa_(2) = −0.25 V versusvs. Fc^+^/Fc, a notable increase in anodic peak current function, *i*_pa_(2)*v* ^−1/2^ plotted against scan rate *v*, was observed at scan rates below 0.2 V s^−1^.^[^ ^78^ ^]^ This behavior is characteristic of a multielectron transfer that is kinetically limited by a subsequent chemical step, as typically seen in an ECE (electrochemical–chemical–electrochemical) mechanism.^[^ ^78^, ^81^ ^]^ The chemical step in this case is most likely a ligand exchange involving N_2_ and the solvent in the W^II^ oxidation state, which is then followed by electrochemical oxidation to yield a W^III^ species (see **Scheme** **6**A).

The oxidation of the [Mo(N_2_)(P5^*Me*^)] (**17**) occurs at a similar potential *E*_pa_(1) = −1.13 versus Fc^+^/Fc as for the tungsten analogue **21** (Figure 7D). In contrast to tungsten(0) complex **21**, the process at *E*_pa_(1) is only partially reversible at scan rates below 0.1 V s^−1^ and at scan rates under 0.5 V s^−1^, an extra irreversible anodic peak appears in the CVs of [Mo(N_2_)(P5^*Me*^)] (**17**) at *E*_pa_(3) (≈−0.7 V versus Fc^+^/Fc). Further oxidation at *E*_pa_(2) is accompanied by another anodic peak at *E*_pa_(4), which vanishes at higher scan rates (Figure 7E). Apart from similar oxidation potentials for tungsten(0) (**21**) and molybdenum(0) complex (**17**), **17** shows no indication of reversibility for the second oxidation at *E*_pa_(2), even at elevated scan rates of *v* = 2 V s^−1^. IR spectroelectrochemical studies of the [Mo(N_2_)(P5^*Me*^)] complex **17** revealed further notable differences compared to its heavier tungsten analogue **21** (Figure 7F). Oxidation of **17** at *E*_pa_(1) resulted in the disappearance of the N_2_ stretch at 1942 cm^−1^; however, unlike in the case of [W(N_2_)(P5^*Me*^)] (**21**), no new species were detected by IR within the 1800 – 2100 cm^−1^ range by oxidizing **17**. The back‐reduction only partially restored the original IR spectrum of [Mo(N_2_)(P5^*Me*^)] (**17**) (Figure 7F).^[^ ^78^ ^]^

The electrochemical and spectroelectrochemical data show that the cationic tungsten(I) and molybdenum(I) dinitrogen pentaPod complexes in NaBPh_4_/THF exhibit different stabilities, although their reduction occurs at similar potentials *E*_pa_(1) = −1.16 V versus Fc^+^/Fc (W^0^ to W^I^) and *E*_pa_(1) = −1.13 V versus Fc^+^/Fc (Mo^0^ to Mo^I^) (see Figure 7A,D). While the monooxidized tungsten species [W(N_2_)(P5^*Me*^)]^+^ appears to be very stable and is easily converted back to the initial [W(N_2_)(P5^*Me*^)] (**21**) complex upon reduction (Figure 7C), the cationic molybdenum analogue [Mo(N_2_)(P5^*Me*^)]^+^ quickly turns into a new species, probably a Mo^I^(THF)^+^ complex (see Scheme 6; Figure 7F). The W^II^(N_2_)^2+^ species, most likely formed during the second oxidation (2) (Figure 7A), is relatively unstable on the millisecond time scale and presumably undergoes N_2_–THF ligand exchange. However, N_2_ coordination in the case of the tungsten complex is observed upon reduction, as indicated by the partial re‐appearance of the N_2_ stretching band (Figure 7C). In contrast, oxidation of the molybdenum complex beyond *E*_pa_(1) leads to more complex behavior, which is likely associated with ligand exchange of N_2_ by THF. The anodic signal at *E*_pa_(3) can therefore be tentatively attributed to the oxidation of the Mo^I^(THF)^+^ species (see Scheme 6).^[^ ^78^ ^]^

### 3.3 Tuning N_2_RR Ability of Mo and W PentaPod Complexes

To further improve the existing catalysts for ammonia production and investigate the effect of different phosphine residues on our pentaPod system, we extended the concept with another pentaPod ligand containing phospholano (Pln) donor groups, P5^*Pln*^ (**14**).^[^ ^57^ ^]^ In this new ligand design, two dimethylphosphine groups of the original pentaPod ligand P5^*Me*^ (**13**) were replaced with phospholano groups, following our previous modifications in tridentate and tripodal ligand systems (cf. Scheme 3).^[^ ^82^, ^83^ ^]^

**Scheme 7:**
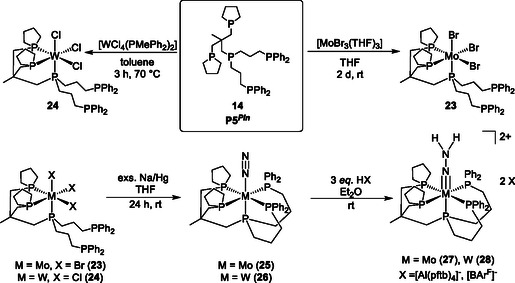
Synthesis of the pentaPod complexes *fac(trpd)*‐[MoBr_3_(P5^*Pln*^)] (**23**) and *fac*(*trpd*)‐[WCl_3_(P5^*Pln*^)] (**24**) bearing the P5^*Pln*^ (**14**) ligand in tripodal fashion was prepared. Mo^0^ (**25**) and W^0^ (**26**) dinitrogen complexes supported by this pentaPod ligand P5^*Pln*^ (**14**) ligand were synthesized by sodium amalgam reduction in THF (exs.: in excess). The N_2_ complexes are treated with three equivalents of acid to generate the corresponding hydrazido(*2‐*) complexes **27** and **28**.^[^ ^57^ ^]^

Originally, phospholanes have been mainly studied in asymmetric catalysis based on chiral bisphospholane ligands,^[^ ^84^, ^85^, ^86^ ^]^ for enantioselective hydrogenation of unsaturated substrates.^[^ ^84^, ^87^ ^]^ The lithium phospholanide (Li‐Pln) is obtained from ethyl dichlorophosphate after a two‐step synthesis.^[^ ^83^ ^]^ To the best of our knowledge, our group was the first to synthesize and isolate Li‐Pln in 2020.^[^ ^83^ ^]^ As a versatile precursor for nonchiral phospholane ligands, Li‐Pln reacts with the compound **12** to afford the product **14** in 85% yield (cf. Scheme 3).^[^ ^57^ ^]^ The dinitrogen complexes [Mo(N_2_)(P5^*Pln*^)] (**25**) and [W(N_2_)(P5^*Pln*^)] (**26**) were synthesized using a procedure analogous to that of the pentaPod system with P5^*Me*^ (**13**) (cf. Scheme 3, **Scheme** **7**). Following the procedure applied for the hydrazido(2‐) complexes bearing the original ligand P5^*Me*^ (**13**), synthesis of [Mo(NNH_2_)(P5^*Pln*^)]X_2_ (**27**) and [W(NNH_2_)(P5^*Pln*^)]X_2_ (**28**) (with X = [BAr^F^]^−^, or X = [Al(pftb)_4_]^−^) were generated by adding 3 equivalents Brookhart`s acid [(Et_2_O)_2_H][BAr^F^] or [H(OEt_2_)_2_][Al(pftb)_4_] to the dinitrogen complexes **25** and **26.** ^[^ ^57^ ^]^

**Figure 8:**
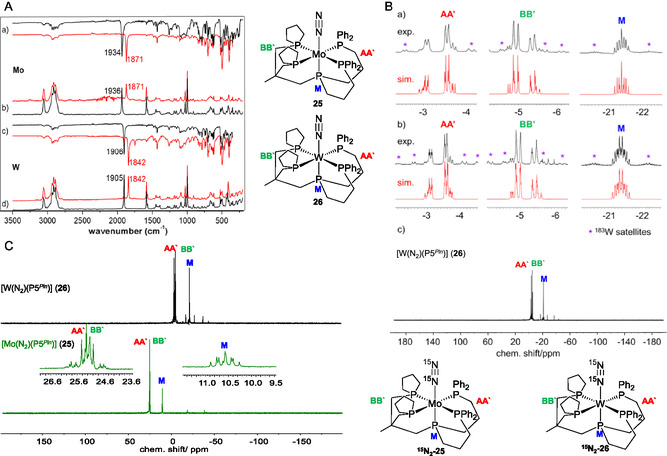
Adapted with permission from ref. [57]. Copyright 2025, Wiley‐VCH. A) IR(ATR) and Raman spectra measured of a,b) [Mo(N_2_)(P5^*Pln*^)] (**25**) (black), ^**15**^ **N‐25** (red), and c,d) [W(N_2_)(P5^*Pln*^)] (**26**) and ^**15**^ **N‐26** (red) showing the band of the N—N stretching vibration; B) experimental ^31^P{^1^H} NMR spectra of a,c) [W(N_2_)(P5^*Pln*^] (**26**) and b) ^**15**^ **N‐26** in benzene‐*d*_6_ at 300 K, compared with the simulation (red); C) experimental ^31^P{^1^H} NMR spectra of [Mo(N_2_)(P5^*Pln*^)] (**25**) (green) and [W(N_2_)(P5^*Pln*^)] (**26**) (black).^[^ ^57^ ^]^

Spectroscopic and NMR analyses were first performed to identify differences in the pentaPod complexes supported by P5^*Me*^ (**13**) and P5^*Pln*^ (**14**). Solid‐state IR (ATR) and Raman spectroscopy reveal N—N stretching bands at 1934 cm^−1^ for [Mo(N_2_)(P5^*Pln*^)] (**25**) and 1906 cm^−1^ for [W(N_2_)(P5^*Pln*^)] (**26**), both of which are 5 cm^−1^ higher in frequency than those observed for the corresponding tungsten (**21**) and molybdenum (**17**) complexes supported by the original pentaPod ligand **13** (**Figure** **8**A; Table 2).^[^ ^57^ ^]^ This means that the N_2_ ligand in the complexes bearing the original pentaPod ligand P5^*Me*^ (**13**) showed a slightly stronger activation. However, these small differences in activation led us to expect reduced catalytic performance and lower ammonia yields with the pentaPod systems **25** and **26**.^[^ ^57^ ^]^ Using the method previously applied to our systems, we investigated the catalytic properties of the complexes bearing the pentaPod ligand P5^*Pln*^ (**14**).^[^ ^60^, ^68^ ^]^ The catalytic ability of [Mo(N_2_)(P5^*Pln*^)] (**25**) and [W(N_2_)(P5^*Pln*^)] (**26**) were studied under the same chemocatalytic conditions as for the systems [Mo(N_2_)(P5^*Me*^)] (**17**) and [W(N_2_)(P5^*Me*^)] (**21**), using 180 equivalents of the PCET reagent SmI_2_(THF)_2_/H_2_O per catalyst (**Table** **1**).^[^ ^57^ ^]^ Complex **25** produced 22.16 ± 0.34 equivalents of ammonia as a catalyst. Although this is slightly less than the 25.73 ± 0.37 equivalents generated by [Mo(N_2_)(P5^*Me*^)] (**17**), the difference can be attributed to the lower activation of **25** compared to **17** (see above, Table 1). Surprisingly, however, the tungsten dinitrogen complex [W(N_2_)(P5^*Pln*^)] (**26**) generated 6.79 ± 0.39 equivalents of ammonia. Despite its slightly lower N_2_ activation compared to [W(N_2_)(P5^*Me*^)] (**21**), [W(N_2_)(P5^*Pln*^)] (**26**) turned out to be the first tungsten complex capable of chemocatalytic N_2_‐to‐NH_3_ conversion.^[^ ^57^ ^]^

**Table 1:** Results of the N_2_‐to‐NH_3_ conversion under chemocatalytic conditions (180 equivalents SmI_2_(THF)_2_/H_2_O, 1 atm N_2_) with pentaPod complexes [M(N_2_)P5^*R*^] (M = mo, *R* = me (**17**), Pln (**25**); M = W, *R* = me (**21**), Pln (**26**)) and in situ generated [M(NNH_2_)P5^*R*^]^2+^ complexes (M = Mo, *R* = Me (**18**), Pln (**27**); M = W, *R* = Me (**22**), Pln (**28**)).^[^ ^60^, ^78^ ^]^

Complex (catalyst)	P5^*R*^	Equivalents NH_3_/M
[Mo(N_2_)(P5^*R*^)]	P5^*Me*^	25.73 ± 0.3
	P5^*Pln*^	22.16 ± 0.34
[Mo(NNH_2_)(P5^*R*^)]^2+^	P5^*Me*^	26.14 ± 0.32
	P5^*Pln*^	22.71 ± 0.62
[W(N_2_)(P5^*R*^)]	P5^Me^	2.75 ± 0.23
	P5^*Pln*^	6.79 ± 0.39
[W(NNH_2_)(P5^*R*^)]^2+^	P5^*Me*^	1.91 ± 0.16
	P5^*Pln*^	7.34 ± 0.37

**Table 2:** Comparison of N‐N stretching vibrations (ν(NN) and ν(^15^N^15^N)) selected bond length from single‐crystal structures of pentaPod complexes [Mo(N_2_)(P5^*Me*^)] (**17**), [Mo(N_2_)(P5^*Pln*^)] (**25**), [W(N_2_)(P5^*Me*^)] (**21**), and [W(N_2_)(P5^*Pln*^)] (**26**).^[^ ^53^, ^57^, ^60^, ^78^ ^]^

	[Mo(N_2_)(P5^*Me*^)] (**17**)^[^ ^53^ ^]^	[Mo(N_2_)(P5^*Pln*^)] (**25**)^[^ ^57^ ^]^	[W(N_2_)(P5^*Me*^)] (**21**)^[^ ^78^ ^]^	[W(N_2_)(P5^*Pln*^)] (**26**)^[^ ^57^ ^]^
N—N bond [Å]	1.099(5)	–	1.084(5)	1.102(6)
M–N_*α*_ bond [Å]	2.033(5)	–	2.020(3)	2.021(4)
M–P_ax_ [Å]	2.3868(12)	–	2.3827(10)	2.3918(11)
ν(NN) [cm^−1^]	1929	1934	1901	1906
ν(^15^N^15^N) [cm^−1^]	1868	1871	1840	1842
^1^ *J*(^15^N_*α*_‐^15^N_β_) [Hz]	6.90	5.99	7.40	6.00

In addition to the dinitrogen complexes, we also investigated the chemocatalytic activity of the corresponding hydrazido(2‐) complexes [Mo(NNH_2_)(P5^*Pln*^)]X_2_ (**27**) and [W(NNH_2_)(P5^*Pln*^)]X_2_ (**28**) (X = [Al(pftb)_4_]^−^). Following our previous approach, complexes **27** and **28** generated the same amount of ammonia as obtained with the corresponding molybdenum and tungsten dinitrogen complexes, within the error limits (Table 1).^[^ ^57^ ^]^

To gain insight into the unexpected catalytic activity of the tungsten complex **26**, and to better understand the general differences in N_2_‐to‐NH_3_ conversion between tungsten and molybdenum, particularly the influence of the pentaPod ligands P5^*Me*^ (**13**) and P5^*Pln*^ (**14**), we examined and compared the complexes [M(N_2_)(L)] with M = Mo (L = P5^*Me*^ (**17**), P5^*Pln*^ (**25**)) and W (L = P5^*Me*^ (**21**), P5^*Pln*^ (**26**)) and corresponding [M(NNH_2_)(L)]^2+^ with M = Mo (L = P5^*Me*^ (**18**), P5^*Pln*^ (**27**)) and W (L = P5^*Me*^ (**22**), P5^*Pln*^ (**28**)) not only by NMR and single‐crystal X‐ray analysis, but also through cyclic voltammetry studies with other electrolytes. Instead of the previously used 20 mM THF/NaBPh_4_,^[^ ^78^ ^]^ we employed THF/Na[BAr^F^] (30 mM) or THF/Li[Al(pftb)_4_] (30 mM) as electrolytes.^[^ ^57^ ^]^

After obtaining a single crystal structure of the complex [W(N_2_)(P5^*Pln*^)] (**26**), a comparison of bond lengths across our pentaPod systems [Mo(N_2_)(P5^*Me*^)] (**17**) and [W(N_2_)(P5^*Me*^)] (**21**) was possible. The bond lengths of the tungsten complex **26** show only minimal differences compared to the bond lengths of the molybdenum **17** and tungsten complexes **21** supported by the original pentaPod ligand P5^*Me*^ (**13**) (see Table 1).^[^ ^53^, ^57^, ^60^ ^]^ In particular, the W–N_*α*_ bond lengths in complexes [W(N_2_)(P5^*Me*^)] (**21**) (2.020(3) Å) and [W(N_2_)(P5^*Pln*^)] (**26**) (2.021(4) Å) are essentially identical. In contrast, a slight elongation of the N—N bond is observed in **26** (1.102(6) Å versus 1.084(5) Å in **21**). Aside from this important difference, the single‐crystal data of [W(N_2_)(P5^*Pln*^)] (**26**) and the tungsten complex **21** bearing the original pentaPod P5^*Me*^ (**13**) exhibit only minor structural variations.^[^ ^57^, ^78^ ^]^

The ^31^P{^1^H} NMR spectrum of the pentaPod ligand P5^*Pln*^ (**14**) revealed a low‐field shift of the P^*Pln*^ signals indicating the phosphorus atom incorporated in the phospholane ring is less electron‐donating compared to the one bearing the two methyl groups of the original pentaPod P5^*Me*^ (**13**). In analogy to our earlier studies on complexes [Mo(N_2_)(P5^*Me*^)] (**17**) and [W(N_2_)(P5^*Me*^)] (**21**), an AA′XX′M coupling pattern was anticipated for the ^31^P{^1^H} NMR spectra of the new complexes with the P5^*Pln*^ ligand **22**. Instead, we observed AA′BB′M coupling patterns for [Mo(N_2_)(P5^*Pln*^)] (**25**) and [W(N_2_)(P5^*Pln*^)] (**26**) (see Figure 8B). A similar low‐field shift of the coordinated P^Pln^ nuclei was observed in both complexes, and in the case of **26**, the chemical shifts of P^AA′^ = −3.42 ppm and P^BB′^ = −5.20 ppm are still distinguishable. In contrast, the **25** exhibits overlapping signals for P^AA′^ = 25.33 ppm and P^BB′^ = 25.20 ppm, making spectral simulation impractical. (see Figure 8C).^[^ ^57^ ^]^

The ^31^P–^31^P coupling constants do not reveal significant differences among the tungsten (**21**, **26**) and molybdenum (**17**, **25**) pentaPod complexes. Detailed NMR‐spectroscopic analysis of the M‐N_2_ unit in the ^15^N‐labeled complexes, however, revealed a decrease of the ^1^ *J*(^15^N_*α*_,^15^N_*β*_) coupling constant for the tungsten P5^*Pln*^ complex [W(^15^N_2_)(P5^*Pln*^)] (^**15**^ **N‐26**, 6.0 Hz) compared to its P5^*Me*^‐supported analog, [W(^15^N_2_)(P5^*Me*^)] (^**15**^ **N‐21**, 7.4 Hz). This indicates a slight weakening of the N—N bond in ^**15**^ **N‐26**, in agreement with the X‐ray structure determination.^[^ ^57^, ^78^ ^]^ The weakening of the N—N bond is also visible for the molybdenum pentaPod complexes (**16**, **25**) based on a decrease of ^1^ *J*(^15^N_*α*_,^15^N_*β*_) from 6.9 Hz (^**15**^ **N‐17**)^[^ ^53^ ^]^ to 5.99 Hz (^**15**^ **N‐25**) (**Table** **2**).^[^ ^53^, ^57^ ^]^

Building on our previous cyclic voltammetry experiments,^[^ ^78^ ^]^ electrochemical studies were extended to complexes [Mo(N_2_)(P5^*Pln*^)] (**25**) and [W(N_2_)(P5^*Pln*^)] (**26**), as well as the hydrazido(2‐) complexes bearing both pentaPod ligands P5^*Me*^ (**13**) and P5^*Pln*^ (**22**) in THF/Na[BAr^F^] (30 mM) and THF/Li[Al(pftb)_4_] (30 mM) as electrolytes. The [BAr^F^]^−^ and [Al(pftb)_4_]^−^ anions were chosen owing to their weaker coordinating ability and reduced redox/basicity relative to [BPh_4_]^−^ from our previous electrochemical study based on the electrolyte THF/Na(BPh_4_).^[^ ^57^, ^78^ ^]^ In THF/Na[BAr^F^] (30 mM), all four complexes exhibited reversible redox events for the M^I^/M^0^ redox couple at −1.17 V for [Mo(N_2_)(P5^*Me*^)] (**17**), −1.19 V for [W(N_2_)(P5^*M*e^)] (**21**), −1.18 V for [Mo(N_2_)(P5^*Pln*^)] (**25**), and −1.19 V for [W(N_2_)(P5^*Pln*^)] (**26**) versus Fc^+^/Fc. The redox potentials are similar to previous ones found in THF/NaBPh_4_ (20 mm) for [Mo(N_2_)(P5^*Me*^)] (**17**) and [W(N_2_)(P5^*Me*^)] (**21**).^[^ ^57^ ^]^ Furthermore, we observed in THF/Na[BAr^F^] (30 mM) an additional reversible event at −1.51 V for molybdenum dinitrogen complexes **17** and **21**, along with new reduction peaks at −1.44 and −1.45 V versus Fc^+^/Fc for the tungsten dinitrogen complexes **20** and **26**, respectively (**Table** **3**).^[^ ^57^ ^]^

**Table 3:** Electrochemical data for N_2_ and NNH_2_ complexes (1 mM) of molybdenum and tungsten with P5^*Me*^ (**13**) and P5^*Pln*^ (**14**) ligands determined from CV measurements at a glassy carbon electrode in THF/Li[Al(pftb)_4_] (30 mm) for *v* = 0.1 V s^−1^.^[^ ^57^ ^]^

	Ligand (L)	Metal	E/V versus Fc^+^/Fc	Redox couple
[M(L)P5^*Me*^]	N_2_	Mo (**17**)	*E*_pa_(1) = −1.17^a^^)^	Mo^I/0^
		W (**21**)	*E*_1/2_(1) = −1.22	W^I/0^
	NNH_2_	Mo (**18**)	*E*_1/2_(2) = −1.49	Mo^IV/III^
		W (**22**)	*E*_1/2_(2) = −1.48	W^IV/III^
[M(L)P5^*Pln*^]	N_2_	Mo (**25**)	*E*_1/2_(1) = −1.21	Mo^I/0^
		W (**26**)	*E*_1/2_(1) = −1.22	W^I/0^
	NNH_2_	Mo (**27**)	*E*_1/2_(2) = −1.47	Mo^IV/III^
		W (**28**)	*E*_1/2_(2) = −1.49	W^IV/III^

a) Irreversible anodic peak.

**Figure 9:**
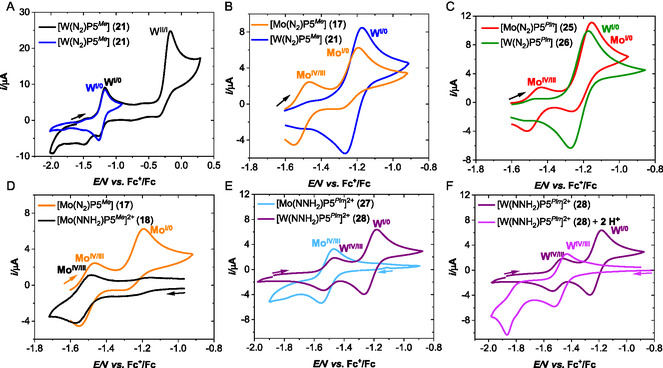
Adapted with permission from ref. [57]. Copyright 2025, Wiley‐VCH. CVs at a glassy carbon working electrode in THF/Li[Al(Opftb)_4_] (30 mM) under argon of A) complex **21** (blue and black) for different potential ranges; B) complexes **17** (orange) and **21** (blue); C) complexes **25** (red) and **26** (green); D) complex **17** (orange) and **18** (black) E) complex **27** (light blue) and complex **28** (purple); F) complex **28** (purple) and complex **28** with two added equivalents of acid ([H(OEt_2_)_2_][Al(pftb)_4_]) (pink). The arrows indicate the initial scanning direction and the starting potential values. Concentration in complex: 1 mM. *E/*V vs.versus Fc^+^/Fc, scan rate *v* = 0.1 V s^−1^.^[^ ^57^ ^]^

Due to decomposition of the corresponding hydrazido(2‐) complexes in THF/Na[BAr^F^], we continued our electrochemistry study in THF/Li[Al(pftb)_4_] (30 mM) as electrolyte (cf. Table 3).^[^ ^57^ ^]^ The CVs in THF/Li[Al(pftb)_4_] of the complexes [W(N_2_)(P5^*Me*^)] (**21**) and [W(N_2_)(P5^*Pln*^)] (**26**) showed the reversible redox event for the W^I/0^ at −1.22 V versus Fc^+/0^ in analogy to THF/Na[BAr^F^] (30 mM). Surprisingly, CVs in the case of the [Mo(N_2_)(P5^*Me*^)] (**17**) and [Mo(N_2_)(P5^*Pln*^)] (**25**) revealed two redox processes in the −1.6 to −1.0 V potential range (**Figure** **9**A–C, orange curve for **17** and red curve for **25**). The first process at *E*_1/2_ (2) = −1.49 V (**17**) and *E*_1/2_ (2) = −1.47 V (**25**) was found to be reversible, whereas the assigned Mo^I/0^ oxidation wave for the molybdenum pentaPod complexes **17** and **25** at *E*_pa_ (1) = −1.17 V versus Fc^+^/Fc is below *v* = 0.1 V s^−1^ irreversible.^[^ ^57^ ^]^

Based on our previous IR spectroelectrochemical studies in THF/Na(BPh_4_),^[^ ^78^ ^]^ the irreversibility of the Mo^I/0^ oxidation wave at −1.13 V versus Fc^+^/Fc is attributed to **N**_**2**_ decoordination at the Mo^I^ state, followed by solvent coordination. The additional reversible redox wave we observed for both molybdenum dinitrogen complexes at *E*_1/2_ (2) = −1.49 V (**17**) and at *E*_1/2_ (2) = −1.47 V (**25**) suggests that the initial complex is converted into a new species. We proposed partial formation of the molybdenum hydrazido(2‐) complexes [Mo(NNH_2_)(P5^*Me*^)]^2+^ (**18**) and [Mo(NNH_2_)(P5^*Pln*^)]^2+^ (**27**) under these electrochemical conditions in THF/Li[Al(pftb)_4_] (30 mM), which was confirmed by voltammetric study of the chemically synthesized hydrazido(2‐) complexes **18** and **27** (Figure 9D). The proton source for the generation of the Mo^IV^(NNH_2_)^2+^ pentaPod complexes (**18**, **27**) could have its origin in traces of water present in the electrolyte. We determined a water content of 12.7 ppm in the THF used for the electrochemical studies by Karl Fischer titration. This approximately equals 1 mM H_2_O in THF, which nearly corresponds to the concentration of the investigated dinitrogen complexes.^[^ ^57^ ^]^

The CV of pentaPod complexes [Mo(NNH_2_)(P5^*Me*^)]^2+^ (**17**) and [Mo(NNH_2_)(P5^*Pln*^)]^2+^ (**27**) only revealed one reversible redox event for the Mo^IV/III^ redox couple, the tungsten analogous of these hydrazido(2‐) complexes [W(NNH_2_)(P5^*Me*^)]^2+^ (**22**) and [Mo(NNH_2_)(P5^*Pln*^)]^2+^ (**28**) exhibited two reversible redox events (Figure 9E,F). These systems corresponded to the W^IV^(NNH_2_)^2+^/W^III^(NNH_2_)^+^ and W^I^(N_2_)^+^/W^0^(N_2_) redox couples at 1.48 V and −1.22 V versus Fc^+^/Fc (**21**) and 1.49 V and −1.22 V versus Fc^+^/Fc (**28**), respectively (Table 3).^[^ ^57^ ^]^

Adding two equivalents of acid reprotonates the W^0^(N_2_) complex, and the corresponding CV shows the W^IV/III^ redox wave of the hydrazido(2‐)complex for both tungsten pentaPod complexes **22** and **28** (Figure 9F). Supported by this data, we found evidence for a higher acidity of the W‐NNH_2_ systems compared to their Mo congeners, which in turn confirms the higher basicity of the Mo‐ compared with the W‐complexes. We further confirmed this by UV/vis studies under similar electrochemical conditions.^[^ ^57^ ^]^ Overall, substituting two dimethylphosphino groups with phospholane has a minimal impact on the redox behavior of both molybdenum and tungsten pentaPod complexes, which is somewhat surprising considering the reduced electron‐donating character the phospholane relative to the methylphosphino group and the unexpected high catalytic activity of the [W(N_2_)(P5^*Pln*^)] (**26**).^[^ ^57^ ^]^

### 3.4 N_2_RR versus HER: Reactivity of [M(N_2_)(P5^*Me*^)] Complexes (M = Mo, W)

Complex [W(N_2_)(P5^*Me*^)] (**21**) was found to primarily catalyze the hydrogen evolution reaction (HER) under N_2_RR conditions (one equivalent complex; 180 equivalents PCET reagent SmI_2_(THF)/H_2_O), in contrast to its molybdenum analogue, which functions as a catalyst for N_2_‐to‐NH_3_ conversion.^[^ ^80^ ^]^

Hydrogen production was quantified by gas‐chromatographic analysis of the headspace above the reaction mixture after the catalysis experiment. The complex [Mo(N_2_)(P5^*Me*^)] (**17**) generated 52.0 equivalents of H_2_ (58% yield based on the reductant) and 25.7 equivalents of NH_3_ (42%). In contrast, for the heavier tungsten analogue **21** 79.7 equivalents of H_2_ (89%) but only 2.8 equivalents of NH_3_ (5%) were detected under these conditions, indicating that nearly all reductant is consumed for HER rather than N_2_RR (see **Table** **4**).^[^ ^80^ ^]^

**Table 4:** Ammonia (N_2_RR) and hydrogen quantities (HER) obtained after reactions with [Mo(N_2_)(P5^*Me*^)] (**17**) and [W(N_2_)(P5^*Me*^)] (**21**) under catalytic conditions (180 equivalents SmI_2_(THF)_2_/H_2_O; 1 atm N_2_).^[^ ^78^ ^]^

Catalyst	Equivalents NH_3_/M	NH_3_ [%]	Equivalents H_2_/M	H_2_ [%]
[Mo(N_2_)(P5^*Me*^)] (**17**)	25.73 ± 0.37^[^ ^60^ ^]^	43	52.02 ± 1.45^[^ ^80^ ^]^	57
[W(N_2_)(P5^*Me*^)] (**21**)	2.75 ± 0.23^[^ ^78^ ^]^	5	79.70 ± 3.81^[^ ^78^ ^]^	89

After experimentally determining the HER activity for the molybdenum(0) (**17**) and tungsten(0) (**21**) pentaPod complexes, we conducted DFT calculations to explore possible mechanisms for both N_2_RR and HER, aiming to determine the origin of their differing reactivities. Specifically, we computed the Gibbs free activation energies for key elementary steps in the catalytic dinitrogen to ammonia conversion and in HER for both complexes [Mo(N_2_)(P5^*Me*^)] (**17**) and [W(N_2_)(P5^*Me*^)] (**21**).^[^ ^80^ ^]^ From a thermodynamic perspective, ammonia formation with complex **17** and **21** can be achieved using either the [M^0^N_2_] or the [M^I^N_2_]^+^ species (M = Mo or W) in the presence of SmI_2_(THF)/H_2_O.^[^ ^60^, ^80^ ^]^

The first PCET step forming the diazenido(‐) intermediate is energetically feasible from both [M^0^N_2_] and [M^I^N_2_]^+^ complexes (M = Mo or W), though slightly endergonic for M^0^ and exergonic for M^I^ (see below).^[^ ^60^ ^]^ As [M^0^N_2_] complexes are used for catalysis, this raises the question of how a catalytic cycle can shift from an M^0^ to an M^I^ pathway. In the M^0^ cycle, the neutral intermediates involve metal oxidation states ranging from 0 to +III, whereas in the M^I^ cycle, the mono‐cationic intermediates the oxidation states range from +I to +IV. The most significant difference between the two pathways lies in the first PCET step: this reaction is endergonic for the M^0^ cycle, with +8.7 kcal mol^−1^ for molybdenum and +6.4 kcal mol^−1^ for tungsten, but becomes strongly exergonic starting from a [Mo^I^N_2_]^+^ complex, giving −19.8 kcal mol^−1^ for Mo and −23.6 kcal mol^−1^ for W.^[^ ^80^ ^]^

**Figure 10:**
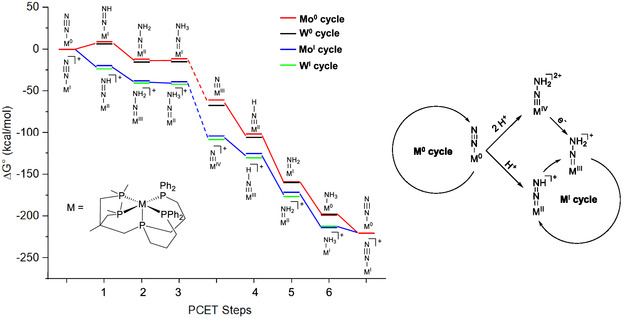
Adapted with permission from ref. [80]. Copyright 2023, Wiley‐VCH. Energy diagram for the N_2_‐to‐NH_3_ conversion via PCET steps, starting from [Mo^0^N_2_] (red)/ [W^0^N_2_] (black) and [Mo^I^N_2_]^+^ (blue)/ [W^I^N_2_]^+^ (green) complexes indicating the N—N bond cleavage step with dashed lines (left); proposed pathways for transitioning from the M^0^ to the M^I^ cycle via protonation of the [Mo^0^N_2_] complex (M = Mo or W) under catalytic conditions (right).^[^ ^80^ ^]^

Overall, the M^I^ cycle is energetically more favorable as it consists entirely of exergonic steps, whereas the N_2_‐to‐NH_3_ conversion over M^0^ pathway includes two slightly endergonic reactions. Except for the initial step, there are no significant energetic differences between the two cycles that would warrant the exclusion of either pathway for the N_2_RR by these pentaPod complexes [Mo(N_2_)(P5^*Me*^)] (**17**) and [W(N_2_)(P5^*Me*^)] (**21**). Moreover, complexes **17** and **21** display nearly identical energetics, suggesting that thermodynamics alone does not account for their differing catalytic behavior (see **Figure** **10**).^[^ ^80^ ^]^

To change from the M^0^ to the M^I^ cycle, an intermediate of the M^I^ pathway must first be accessed. Starting from the [M^0^N_2_] complex, potential entry points include the [M^II^NNH]^+^ and [M^III^NNH_2_]^+^ species. These could be accessed via monoprotonation yielding [M^II^NNH]^+^, a known intermediate of the M^0^ cycle, or deprotonation to form [M^IV^NNH_2_]^2+^, followed by single‐electron reduction (Figure 10).^[^ ^80^ ^]^ This requires that the PCET reagent SmI_2_(THF)/H_2_O, or the Sm^III^ species formed after a PCET step, is capable of independently transferring protons or electrons, in addition to functioning as a PCET reagent. In our DFT study, instead of the distal mechanism, we also considered an alternating mechanism starting from M^0^ and a hybrid mechanism for the N_2_‐to‐NH_3_ conversion. The alternative pathway for the M^0^ cycle displays exclusively exergonic steps, in contrast to the distal pathway, which includes two slightly endergonic steps. Therefore, an alternating pathway for N_2_RR in the M^0^ cycle seems to be preferred. All three mechanisms for N_2_RR are thermodynamically possible for the M^I^ cycle as well. Overall, a hybrid mechanism including a [M^II^(NHNH_2_)]^+^ species shows the most exergonic pathway for the M^I^ cycle. This is further supported by the computed N—H BDFE for various M–N_*x*_H_*y*_ species (*x* = 1–2, *y* = 1–4) across both catalytic cycles corresponding to the distal and alternating pathways (**Table** **5**). However, the actual course for N_2_RR for both cycles is also influenced by kinetic factors. In this context, PCET reactions at the distal nitrogen of the N_2_ ligand are generally preferred due to steric factors.^[^ ^80^ ^]^

**Table 5:** Calculated N—H BDFE of terminal N—H bonds for different M–N_*x*_H_*y*_ intermediates (M = Mo, W) of both discussed cycles for [Mo(N_2_)(P5^*Me*^)] (**17**) and [W(N_2_)(P5^*Me*^)] (**21**) along the distal (D) and alternating (A) pathway.^[^ ^80^ ^]^

Pathway	M^0^ cycle	N—H BDFE [kcal mol^−1^]		M^I^ cycle	N—H BDFE [kcal mol^−1^]	
		Mo	W		Mo	W
D + A	[M^I^NNH]	17.3	19.6	[M^II^NNH]^+^	45.8	49.6
D	[M^II^NNH_2_]	46.8	47.9	[M^III^NNH_2_]^+^	43.9	42.8
D	[M^I^NNH_3_]	25.5	25.5	[M^II^NNH_3_]^+^	27.1	27.6
A	[M^0^NHNH]	53.3	52.3	[M^I^NHNH]^+^	75.0	76.5
A	[M^I^NHNH_2_]	55.2	56.0	[M^II^NHNH_2_]^+^	67.0	68.9
–	[M^0^NHNH_3_]	38.7	37.0	[M^I^NHNH_3_]^+^	38.2	38.3
A	[M^0^NH_2_NH_2_]	65.8	62.5	[M^I^NH_2_NH_2_]^+^	59.3	54.5
D + A	[M^II^NH]	66.7	64.9	[M^III^NH]^+^	47.3	47.8
D + A	[M^I^NH_2_]	83.2	79.8	[M^II^NH_2_]^+^	72.3	72.1
D + A	[M^0^NH_3_]	66.4	63.9	[M^I^NH_3_]^+^	68.4	61.9

**Scheme 8:**
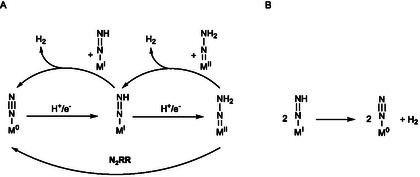
A) Adapted with permission from ref. [80]. Copyright 2023, Wiley‐VCH. Possible reaction pathways for HER from early M–N_*x*_H_*y*_ intermediates in the M^0^ cycle (M = Mo, W) via bimolecular reactions; B) bimolecular reaction of [M^I^(NNH)] (M = Mo, W) yields two equivalents of [Mo^0^(N_2_)] complex and molecular hydrogen.^[^ ^80^ ^]^

With respect to HER, we examined two possible pathways.^[^ ^88^, ^89^ ^]^ In the literature, two primary pathways are commonly discussed: i) bimolecular reactions involving early M–N_*x*_H_*y*_ intermediates (**Scheme** **8**), and ii) hydrogen formation via metal hydride species.^[^ ^78^ ^]^ In the Mo^0^ cycle, reactions of early M–N_*x*_H_*y*_ intermediates are more exergonic than the corresponding N_2_RR steps. After the first equivalent of ammonia is released, all bimolecular reactions involving late‐stage M–N_*x*_H_*y*_ intermediates in the M^0^ cycle become endergonic, consistent with the calculated N—H BDFE's (cf. Table 5). In contrast, bimolecular reactions of early M–N_*x*_H_*y*_ intermediates in the M^I^ cycle are less favorable compared to the M^0^ cycle, as N_2_RR is more accessible in the presence of excess PCET reagent. Formation of the intermediate [M^II^(NHNH_2_)]^+^ along the alternating pathway for N_2_RR is the most exergonic process and is also expected to be kinetically more favorable than the competing PCET step leading to [M^IV^(H)(NNH_2_)]^+^, which could release H_2_ via a monomolecular process. However, HER via bimolecular reactions revealed no significant difference between complexes [Mo(N_2_)(P5^*Me*^)] (**17**) and [W(N_2_)(P5^*Me*^)] (**21**).^[^ ^80^ ^]^

Alternatively, HER catalyzed by [Mo(N_2_)(P5^*Me*^)] (**17**) and [W(N_2_)(P5^*Me*^)] (**21**) could also proceed via hydride complexes. For the M^0^ cycle we considered that the formation of a [M^I^H] complex could serve as the starting point of HER. HER via the hydride pathway is less favorable for W, as its associated energy is ≈7 kcal mol^−1^ higher than for Mo. Thus, this pathway alone does not account for the differences in HER activity between the two metals.^[^ ^80^ ^]^ Similar observations were made in the M^I^ cycle. The dissociative mechanism, in which N_2_ dissociation constitutes the first step toward [M^II^H] formation, is highly unlikely due to the strongly endergonic release of the N_2_ ligand. Subsequent H_2_ release from the [Mo^III^(H)_2_] species is, however, significantly less endergonic than H_2_ release from the corresponding [M^II^(H)_2_] species in the M^0^ cycle.^[^ ^80^ ^]^

HER in the M^I^ cycle via early M–N_*x*_H_*y*_ intermediates through an associative mechanism is likely similar for both metals, consistent with observations in the M^0^ cycle. In summary, HER with our systems [Mo(N_2_)(P5^*Me*^)] (**17**) and [W(N_2_)(P5^*Me*^)] (**21**) via hydride species is consistently endergonic and thus thermodynamically less favorable. Steric hindrances slow PCET at the metal center compared to reactions at the nitrogen atom of the N_2_ ligand. In contrast, HER via mixed [M(H)(N_*x*_H_*y*_)] species is highly exergonic, especially within the neutral M^0^ intermediates, suggesting these are more favorable starting points.^[^ ^80^ ^]^

To favor N_2_RR over HER with our pentaPod complexes, reaction conditions should be chosen that prevent entry into the M^0^ cycle by avoiding over‐reduction. Therefore, PCET reagents with appropriate redox potentials should be employed. Our DFT calculations do not indicate significant differences between molybdenum and tungsten. The observed reactivity differences between Mo and W systems thus may derive from kinetic barriers in PCET steps along the N_2_RR pathway, which were not explored theoretically so far.^[^ ^80^ ^]^

## 4 Summary and Outlook

The pentaPod concept containing the ligands P5^*Me*^ (**13**) and P5^*Pln*^ (**14**) provides a versatile platform for detailed studies of N_2_ activation and N_2_‐to‐NH_3_ conversion with Chatt‐type molybdenum(0) and tungsten(0) dinitrogen complexes. The strong chelating ability of pentaPod ligands **13** and **14** generates an inert, yet flexible coordination environment that enables protonation and reduction of N_2_ in these complexes while preserving the pentaphosphine framework. This enabled not only the preparation of complexes [M(N_2_)(P5^*R*^)], but also the synthesis and analysis of the corresponding hydrazido(*2‐*)complexes [M(NNH_2_)(P5^*R*^)]^2+^ (M = Mo and W; *R* = Me, Pln).^[^ ^53^, ^57^, ^78^ ^]^

Catalytic studies of [Mo(N_2_)(P5^*Me*^)] (**17**) yielded 26 equivalents of NH_3_ using 180 equivalents of SmI_2_(THF)_2_/H_2_O as the PCET reagent. The NN stretching frequency of **17** (ν(NN) = 1929 cm^−1^) is the lowest of all molybdenum complexes with pentaphosphine ligation.^[^ ^60^ ^]^ Replacement of the methylphosphino groups by phospholanes led to the [Mo(N_2_)(P5^*Pln*^)] (**25**) complex (ν(NN) = 1934 cm^−1^), which produced 22 equivalents of NH_3_ under identical conditions.^[^ ^57^ ^]^ Notably, the [W(N_2_)(P5^*Pln*^)] complex (**26**) (ν(NN) = 1906 cm^−1^) also generated catalytic amounts of ammonia (7 equivalents), rendering it the first tungsten complex to exhibit chemocatalytic N_2_‐to‐NH_3_ conversion. Although the [W(N_2_)(P5^*Me*^)] complex (**21**) shows a higher degree of N_2_ activation compared to **26**, as indicated by a ν(NN) = 1901 cm^−1^, it produces only 3 equivalents of ammonia.^[^ ^57^, ^78^ ^]^ This finding also demonstrates that the degree of N_2_ activation is not necessarily indicative of a complex’ catalytic performance in N_2_‐to‐NH_3_ conversion. As demonstrated by these results, the design of the pentaPod ligands compensates for the inherent drawbacks of classic Chatt complexes. Moreover, the inability of the pentaPod complexes to dimerize supports the mononuclear PCET pathway within the Chatt cycle for the catalytic generation of ammonia.^[^ ^57^, ^60^, ^78^ ^]^

Although the different (chemo‐)catalytic performance of tungsten and molybdenum complexes in N_2_RR and the competing HER have been described in the literature, the underlying cause has not yet been fully understood.^[^ ^57^ ^]^ The pentaPod concept thus provides an ideal platform for investigating the differences between molybdenum and tungsten complexes and their ability to catalyze N_2_‐to‐NH_3_ conversion.

Single‐crystal structure determination shows only minor structural differences between the molybdenum(0) and tungsten(0) dinitrogen pentaPod complexes. Similarly, NMR spectroscopy reveals only slight differences among the pentaPod complexes, apart from the fact that the ^31^P{^1^H} NMR spectra display an AA′XX′M splitting pattern for [Mo(N_2_)(P5^*Me*^)] (**17**) and [W(N_2_)(P5^*Me*^)] (**21**) whereas complexes [Mo(N_2_)(P5^*Pln*^)] (**25**) and [W(N_2_)(P5^*Pln*^)] (**26**) exhibit AA′BB′M patterns. This change indicates a variation of the electronic structure caused by the phospholano substituent of **25** and **26** compared to **17** and **21**. However, this does not entail significant structural differences between molybdenum and tungsten pentaPod complexes or any obvious electronic effect that can be directly attributed to the PMe_2_ or phospholano substituents of the pentaPod ligands **13** and **14**.

In order to obtain more information on that issue, the electrochemical properties and redox behavior of the pentaPod complexes were investigated by CV and IR spectroelectrochemistry. The tungsten(0) (**21**) and molybdenum(0) dinitrogen (**17**) complexes undergo in THF/Na(BPh_4_) (20 mM) one‐electron oxidation at almost identical potentials, *E*_pa_ = −1.16 and −1.13 V versus Fc^+^/Fc, respectively. IR spectroelectrochemistry revealed stability differences between these pentaPod complexes **17** and **21**. Upon oxidation of [W(N_2_)(P5^*Me*^)] (**21)**, a stable [W^I^(N_2_)(P5^*Me*^)]^+^ complex forms, whereas the molybdenum analogue [Mo(N_2_)(P5^*Me*^)] (**17**) loses N_2_, likely accompanied by ligand exchange and formation of a [Mo^I^(THF)(P5^*Me*^)]^+^ species. For tungsten, N_2_ loss occurs only after further oxidation of complex [W^I^(N_2_)(P5^*Me*^)]^+^, while the analogous Mo^I^ species is unstable.

Our studies were then extended to [Mo(N_2_)(P5^*Pln*^)] (**25)** and [W(N_2_)(P5^*Pln*^)] (**26)** as well as the hydrazido(2‐) complexes [Mo(NNH_2_)(P5^*R*^)]^2+^ (**18**, **27**) and [W(NNH_2_)(P5^*R*^)]^2+^ (**22**, **28**) with *R* = Me, Pln, using THF/Li[Al(pftb)_4_] (30 mM) as electrolyte. Cyclic voltammetry of these systems in THF/Li[Al(pftb)_4_] shows similar redox potentials for all Mo^I/0^ and W^I/0^ couples of the N_2_‐complexes (*E*_1/2_ (1) = −1.21 V versus Fc^+^/Fc) as well as Mo^IV/III^ and W^IV/III^ couples of the corresponding NNH_2_ species (*E*_1/2_(2) = −1.49 V versus Fc^+^/Fc), indicating the redox processes to be largely independent of the type of pentaPod ligand and metal. The latter is remarkable in view of the fact that usually relatively large shifts toward more negative potentials are observed upon going from molybdenum to analogous tungsten systems, also for nitrogenase.^[^ ^90^, ^91^ ^]^ However, by using the supporting electrolyte Li[Al(pftb)_4_], an unexpected voltammetric response was observed for the Mo^0^(N_2_) species, which is attributed to acid–base reactions under the applied electrochemical conditions.^[^ ^57^ ^]^

In the conversion of Mo^0^(N_2_) to Mo^IV^(NNH_2_)^2+^ observed in the CVs, the protons required for this reaction can be obtained from residual water in the electrolyte, which corresponds to ≈1 equiv. of water/complex. As the formation of hydrazido(‐2) complexes was not observed in the CV data of the tungsten(0) dinitrogen complexes **21** and **26**, the Mo^0^(N_2_) complexes **17** and **25** are more basic than their W^0^(N_2_) analogs, regardless of the coordinated pentaPod ligand. These findings thus lead to the surprising conclusion that tungsten dinitrogen complexes such as **21** and **26**, although exhibiting *lower* N—N stretching frequencies than their molybdenum analogues, are less susceptible to protonation than the latter. This could, at least in part, explain the different reactivity between molybdenum(0) and tungsten(0) dinitrogen complexes regarding N_2_RR.^[^ ^57^ ^]^

Finally, our DFT studies leave the possibility that aside from different acidities of Mo and W pentaPod complexes, differences in reactivity may also arise from kinetic barriers associated with PCET steps along the N_2_RR pathway, for which no theoretical data were obtained so far.^[^ ^80^ ^]^

**Scheme 9:**
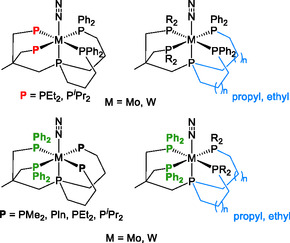
Variation possibilities on the pentaPod ligand scaffold in the trident or tripodal part.

To conclude, based on the pentaPod concept, a series of molybdenum and tungsten dinitrogen complexes have been synthesized that both catalyze the N_2_‐to‐NH_3_ conversion and allow the isolation of well‐defined intermediates along this pathway. In our current work, we are also focusing on isolating further intermediates in the conversion of N_2_ to NH_3_. Owing to its unique topology, we anticipate the pentaPod ligand is able to stabilize these intermediates (see Scheme 1; Figure 5) and thereby enable their synthetic isolation. In addition, the pentaPod concept offers a wide range of possibilities for structural variations (**Scheme** **9**).

The pentaPod concept offers considerable flexibility, that is, the substituents in the equatorial plane and the tripodal as well as the trident framework of the pentaPod ligands can be easily modified, enabling systematic variation of alkylphosphino donors and assessment of their effects on N_2_ activation and catalytic N_2_‐to‐NH_3_ conversion (see Scheme 9). As the steric properties of the substituents appear to also influence catalytic performance, not only the tripodal unit, but also the overall ligand scaffold can be tuned. Adjustments to the ligand backbone or the length of the alkyl chains present additional opportunities for optimization and investigation. Specifically, the tridentate part of the pentaPod ligand can be altered by introducing ethyl or butyl linkers, which may impact both the flexibility of the system and the coordination behavior of the P_ax_ donor trans to the N_2_ ligand.

## Conflict of Interest

The authors declare no conflict of interest.
